# Hinge-Region O-Glycosylation of Human Immunoglobulin G3 (IgG3)* [Fn FN2]

**DOI:** 10.1074/mcp.M114.047381

**Published:** 2015-03-10

**Authors:** Rosina Plomp, Gillian Dekkers, Yoann Rombouts, Remco Visser, Carolien A.M. Koeleman, Guinevere S.M. Kammeijer, Bas C. Jansen, Theo Rispens, Paul J. Hensbergen, Gestur Vidarsson, Manfred Wuhrer

**Affiliations:** From the ‡Center for Proteomics and Metabolomics,; §Department of Rheumatology, Leiden University Medical Center, Leiden, The Netherlands;; ¶Department of Experimental Immunohematology,; ¶¶Department of Immunopathology, Sanquin Research, and Landsteiner Laboratory, Academic Medical Center, University of Amsterdam, Amsterdam, The Netherlands;; **Division of BioAnalytical Chemistry, VU University Amsterdam, Amsterdam, The Netherlands

## Abstract

Immunoglobulin G (IgG) is one of the most abundant proteins present in human serum and a fundamental component of the immune system. IgG3 represents ∼8% of the total amount of IgG in human serum and stands out from the other IgG subclasses because of its elongated hinge region and enhanced effector functions. This study reports partial O-glycosylation of the IgG3 hinge region, observed with nanoLC-ESI-IT-MS(/MS) analysis after proteolytic digestion. The repeat regions within the IgG3 hinge were found to be in part O-glycosylated at the threonine in the triple repeat motif. Non-, mono- and disialylated core 1-type O-glycans were detected in various IgG3 samples, both poly- and monoclonal. NanoLC-ESI-IT-MS/MS with electron transfer dissociation fragmentation and CE-MS/MS with CID fragmentation were used to determine the site of IgG3 O-glycosylation. The O-glycosylation site was further confirmed by the recombinant production of mutant IgG3 in which potential O-glycosylation sites had been knocked out.

For IgG3 samples from six donors we found similar O-glycan structures and site occupancies, whereas for the same samples the conserved N-glycosylation of the Fc CH2 domain showed considerable interindividual variation. The occupancy of each of the three O-glycosylation sites was found to be ∼10% in six serum-derived IgG3 samples and ∼13% in two monoclonal IgG3 allotypes.

Immunoglobulin G (IgG) is one of the most abundant proteins present in human serum and represents approximately three-quarters of the total serum immunoglobulin content (1). As the main mediator of humoral immunity and an important link between the adaptive and innate immune system, IgG is a fundamental component of the immune system. IgG consists of two heavy and light chains, linked by disulfide bonds. The protein can be subdivided into the antigen-binding (Fab) and the receptor-binding (Fc) region. There are four subclasses of IgG, all of which share an overall structure homology but differ slightly in their amino acid sequence; the quantity of the subclasses in human serum is as follows: IgG1 > 2 > 3 > 4 (2).

IgG3 represents ∼8% of the total amount of IgG in human serum (2), and stands out from the other IgG subclasses for a number of reasons. First of all, IgG3 contains an elongated hinge region with up to a triple repeat sequence (the actual number ranging from one to three depending on the allotype (3)), which is responsible for the increased flexibility between the Fab and the Fc part, as well as the wider and more flexible angle between the two Fab arms (4, 5). This flexibility is likely the cause of the increased affinity of IgG3, compared with the other subclasses, for divalent binding to certain types of antigens (4, 6, 7). Second, IgG3 has a higher affinity for C1q, which initiates the classical complement pathway (5, 8). The interaction between IgG3 and C1q is not due to the elongated hinge region, as demonstrated by studies showing that recombinant IgG3 with an IgG1- or IgG4-like hinge sequence exhibited even greater binding affinity for C1q than wild-type IgG3 (8–10). Third, IgG3 has a higher overall affinity for the Fcγ receptors (FcγRs), through which it can influence effector cells of the innate immune system (11). The CH2 domain and hinge region of IgG3 were shown to be instrumental in binding to the high affinity FcγRI receptor (12). Finally, IgG3 generally has a shorter half-life compared with the other IgG subclasses (1 *versus* 3 weeks) (2). This difference was traced back to an H435R mutation that confers a positive charge at physiological pH, resulting in a decreased binding to the neonatal Fc receptor (FcRn), which is involved in recycling IgG targeted for lysosomal degradation (13). The low-efficiency FcRn-mediated transport also gives rise to decreased levels of IgG3 in mucosal tissue and impaired transport of IgG3 across the placenta (14). These properties do not hold true for all types of IgG3 since a large number of IgG3 allotypes have been described, some of which lack the H435R substitution and have a half-life and placental transport rates similar to IgG1 (13–16). IgG3 is more polymorphic than the other IgG subclasses, as evidenced by the high number of known allotypes (16). Most of the polymorphisms reside in the CH2 or CH3 domain, but the length of the hinge region can also display a high degree of variation. Depending on the number of sequence repeats, the hinge region can vary from 27 to 83 amino acid residues between different IgG3 allotypes (3, 16, 17).

An *N*-linked complex type glycan is highly conserved and found in the CH2 domain of all IgG subclasses and allotypes. The type of glycan present at this site has been shown to influence the effector functions of IgG (18). N-glycans that lack a core fucose cause IgG to have an enhanced proinflammatory capacity through stronger binding to FcγRIIIa and FcγRIIIb (18–20). In contrast, IgG carrying sialylated N-glycans exhibits anti-inflammatory properties, likely due to increased binding affinity to C-type lectins and/or reduced binding to FcγR (18, 21, 22).

O-linked glycosylation has been reported for various immunoglobulins. O-glycans are present on the hinge region of human IgA1 and IgD and mouse IgG2b (23–25). IgA1 contains nine potential sites for O-glycosylation (serine and threonine) in the hinge region, of which 3–5 are occupied, while IgD has been reported to carry between four and seven O-glycans (24–26). The O-glycosylation in the hinge of murine IgG2b was observed to protect against proteolytic digestion (23). Likewise, IgA1 was found to be more susceptible to degradation by *Streptococci* proteases after neuraminidase treatment (27).

In this study, we report partial O-glycosylation of the human IgG3 hinge. We obtained both poly- and monoclonal IgG3 from various sources and performed proteolytic digestion with trypsin or proteinase K. NanoLC-reverse phase (RP)-ESI-ion trap (IT)-MS/MS was used to examine the resulting (glyco)peptides, revealing core 1-type O-glycans on multiple sites within the IgG3 hinge region.

## EXPERIMENTAL PROCEDURES

### 

#### 

##### Immunoglobulin Sources

##### Polyclonal IgG3 from Donor Sera

IgG3 was purified from six serum samples (average donor age: 44.5 ± 9 years; three male and three female; details are listed in supplemental Table S1). The serum samples were collected from healthy donors with informed consent in compliance with the institutional ethical board. Venous blood was collected in a 9-ml Vacuette serum clot activator tube (Greiner BioOne, Kremsmünster, Austria) and incubated at room temperature for 30 min, followed by centrifugation for 15 min at 1800 g. The serum fraction was then collected and stored at −20 °C. IgG was isolated by running the serum over a HiTrap Protein G HP column (GE Healthcare, Buckinghamshire, UK) and eluted with 0.1 m glycine-HCl, pH 2.7. The eluate containing IgG was then applied to a HiTrap MabSelect SuRE column packed with recombinant Protein A (GE Healthcare), and the IgG3-containing flow-through was concentrated using an Amicon Ultra-15 centrifugal filter device 10 kDa (Merck Millipore, Darmstadt, Germany) and dialyzed against PBS using a Slide-A-Lizer Dialysis Cassette, 10K MWCO (Dionex/Thermo Scientific, Sunnyvale, CA).

##### Polyclonal IgG3 from Pooled Plasma

IgG3 purified from pooled plasma was obtained commercially from Athens Research and Technology (Athens, GA). Upon inquiry, we learned that this IgG3 had been treated with dextran sulfate, followed by centrifugation to pellet the lipids. The delipidated plasma was then subjected to an ammonium sulfate precipitation, followed by boric acid precipitation. The IgG was isolated with ion exchange chromatography, and further purification of IgG3 was achieved with affinity chromatography (rProtein A), gel filtration chromatography, and a jacalin column to remove a minor IgA contaminant. This purification method did not expose the IgG3 to extreme pH conditions or temperatures above 55 °C.

##### Monoclonal IgG3

Two monoclonal recombinant anti-GDob1 IgG3 allotypes, G3m(g) and G3m(s), were produced in an HEK-293F FreeStyle cell line expression system (Life Technologies, Paisley, UK). IgG3m(s) was then purified with a HiTrap MabSelect SuRE column packed with recombinant Protein A (GE Healthcare), while IgG3m(g) was purified with a Protein G HiTrap HP column (GE Healthcare).

##### Monoclonal IgG3 Mutants

Three IgG3 G3m(g) hinge mutants were produced recombinantly, together with wild-type IgG3 G3m(g) as a control. The variable regions of the heavy and light chains (VH, VL) of the mouse IgG1 anti-2,4,6-trinitrophenol (TNP) hapten antibodies were cloned onto human IgG3 and kappa backbones, respectively, as described previously (20, 28). Synthetic DNA encoding for IgG3 mutants, replacing the three threonines (T) and/or serines (S) with alanines (A), was generated as 5′-NheI and 3′Bsu36I fragments by GeneArt (Invitrogen). The NheI-Bsu36I fragments were ligated in anti-TNP IgG3 heavy chain replacing the corresponding fragment in the wild-type heavy chain. Antibodies were produced in the HEK-293F FreeStyle cell line expression system (Invitrogen) with cotransfection of vectors encoding p21, p27, and pSVLT genes as described (29) to increase protein production. The antibodies were purified on a protein G HiTrap HP column (GE Healthcare) using the ÄKTAprime plus system (GE Healthcare) and dialyzed against PBS overnight.

##### Monoclonal IgG3/4 Fc Constructs

Four recombinant IgG3 and IgG4 Fc constructs were produced in a HEK-293F FreeStyle cell line (Invitrogen) and purified with a Protein G column (Protein G Sepharose 4 fast flow; GE Healthcare), as described by Rispens *et al.* (30). All constructs contained an IgG4 hinge region, together with either the CH2 and CH3 regions of two IgG3 allotypes (G3m(b)-Fc-h4 and G3m(c3c5)-Fc-h4) or the CH2 and CH3 regions of two IgG4 variants (IgG4-Fc-V397M and IgG4-Fc-V397M,K392N).

##### Polyclonal and Monoclonal IgG4

Several IgG4 samples were collected. A polyclonal IgG4 sample was affinity-purified from the plasma of a rheumatoid arthritis patient using anti-IgG4 coupled to Sepharose (clone MH164.4, Sanquin, Amsterdam, The Netherlands). A second IgG4 sample was enriched from the serum of a patient from the Leiden University Medical Center with an extremely high IgG4 serum titer (14.4 mg/ml). The serum was heat-inactivated and subjected to a 33% cut ammonium sulfate precipitation, followed by dialysis to decrease the salt content. The IgG4 was then enriched on a HiTrap DEAE Sepharose and a Superdex 200 column (GE Healthcare) using an ÄKTAprime plus chromatography system (GE Healthcare); IgG4-containing fractions were identified using ELISA and pooled. A monoclonal anti-TNP IgG4 sample produced in HEK cells was purified with a protein G column and dialyzed against PBS in the same way as described for the IgG3 mutants. In addition, we obtained a recombinant humanized IgG4 sample in the form of the therapeutic antibody natalizumab (Tysabri; Biogen Idec, Badehoevedorp, The Netherlands), which is produced in murine myeloma cells.

The protein sequences of the recombinant samples can be found in supplemental Table S2.

##### SDS-PAGE Analysis and Proteolytic Digestion

Five μg of the IgG samples was reduced with 2-beta-mercaptoethanol (Sigma-Aldrich, St. Louis, MO) at 95 °C for 10 min. The IgG was then run on a NuPage 4–12% Bis-Tris SDS-PAGE gel (Invitrogen) and stained with Coomassie G-250 (SimplyBlue SafeStain, Invitrogen). Bands were excised, cut into pieces, washed with 25 mm ammonium bicarbonate (ABC^1^, Sigma-Aldrich), and dehydrated with acetonitrile (Biosolve, Valkenswaard, The Netherlands). The IgG was then again treated with a reducing agent by adding 50 μl of a 10 mm dl-dithiothreitol (DTT; Sigma-Aldrich) 25 mm ABC solution for 30 min at 55 °C. The gel pieces were subsequently dehydrated by the addition of acetonitrile. Alkylation of the cysteine residues was achieved by incubation with 50 μl of a 55 mm iodoacetamide (Sigma-Aldrich) 25 mm ABC solution in the dark for 20 min. The gel pieces were then washed with 25 mm ABC and dehydrated with acetonitrile. The washing and dehydration was repeated a second time, and the samples were subsequently dried down completely in a centrifugal vacuum concentrator (Eppendorf, Hamburg, Germany).

For proteolytic digestion to take place, 30 μl of 25 mm ABC containing either trypsin (sequencing grade modified trypsin, Promega, Madison, WI), proteinase K (from *Tritirachium album*; Sigma-Aldrich) or chymotrypsin (sequencing grade from bovine pancreas, Roche Applied Sciences, Mannheim, Germany) was added to the dried gel pieces. An IgG:enzyme (w/w) ratio of 1:20 for trypsin, 1:3 for proteinase K and 1:20 for chymotrypsin was used. The samples were kept on ice for 1 h, to allow the enzyme to enter the gel pieces. If the gel pieces were not fully submerged, a further 10–20 μl of 25 mm ABC was added. The samples were incubated overnight at 37 °C. The solution surrounding the gel pieces was collected the next morning. Following the addition of another 20 μl of 25 mm ABC, the gel pieces were incubated at 37 °C for 1 h. The solution was then again collected and added to the first fraction, and stored at −20 °C.

Alternatively, several IgG3 samples were digested in solution without reduction alkylation: 3 μg of IgG3 was incubated with 0.3 μg of trypsin in a total volume of 25 μl 25 mm ABC at 37 °C overnight.

Endoproteinase AspN (New England Biolabs, Ipswich, MA) was used to further digest trypsin-generated (glyco)peptides. Digestion was performed by adding 1.5 μl of AspN and 15 μl of 2x AspN buffer (100 mm Tris-HCl, 5 mm zinc sulfate, New England Biolabs) to 15 μl of trypsin-digested IgG3, and incubating overnight at 37 °C.

##### Exoglycosidase Digestion

Exoglycosidase digestion was performed on tryptic IgG glycopeptides. The trypsin-digested IgG3 was first heated to 95 °C for 5 min to inactivate the trypsin. The sample was then dried in a centrifugal vacuum concentrator (Eppendorf), and resuspended by the addition of 16 μl of Milli-Q-purified water, 2 μl 50 mm sodium acetate (pH 5.5), 1 μl sialidase (Glyko sialidase A, Prozyme, Hayward, CA), and 1 μl of galactosidase (Glyko beta-galactosidase, Prozyme). The samples were incubated overnight at 37 °C.

##### NanoLC-ESI-IT-MS(/MS) Analysis

The IgG3 (glyco)peptides were analyzed with nanoLC-reversed phase (RP)-electrospray (ESI)-ion trap (IT)-MS(/MS) on an Ultimate 3000 RSLCnano system (Dionex/Thermo Scientific) coupled to an amaZon speed ESI-IT-MS (Bruker Daltonics, Bremen, Germany). A precolumn (Acclaim PepMap C18 capillary column, 300 μm x 5 mm, particle size 5 μm, Dionex/Thermo Scientific) was used to wash and concentrate the sample, and separation was achieved on an Acclaim PepMap RSLC C18 nanocolumn (75 μm x 150 mm, particle size 2 μm, Dionex/Thermo Scientific) with a flow rate of 500 nl/min. The following linear gradient was used, with solvent A consisting of 0.1% formic acid in water and solvent B of 95% acetonitrile, 5% water: t = 0 min, 1% solvent B; t = 5 min, 1% B; t = 20 min, 25% B; t = 25 min, 70% B; t = 30 min, 70% B; t = 31 min, 1% B; t = 55 min, 1% B. The sample was ionized in positive ion mode with an ESI-nanosprayer (4500 V) using a bare fused silica capillary (internal diameter of 20 μm). The solvent was evaporated at 180 °C with a nitrogen flow of 5 liters/min. A CaptiveSpray nanoBooster (Bruker Daltonics) was mounted onto the mass spectrometer and saturated the nitrogen flow with ACN to enhance the sensitivity. The MS1 ion detection window was set at *m/z* 350–1400, and the MS2 window at *m/z* 140–2200. The three highest nonsingly charged peaks in each MS1 spectrum were automatically fragmented through collision-induced dissociation (CID). In order to identify the peptide sequence of proteinase K- and trypsin-generated O-glycopeptides, MS3 analysis was performed on manually selected precursors: the MS2 peak representing the peptide without sugars attached was targeted for fragmentation. In a separate LC-MS run, electron transfer dissociation fragmentation was done on selected precursor ions.

##### t-ITP-CE-ESI-qTOF-MS(/MS) Analysis

Transient isotachophoresis (ITP)-capillary electrophoresis (CE)-ESI-qTOF-MS/MS analysis was performed on a tryptic sialidase- and galactosidase-treated IgG3 sample derived from pooled plasma. Separation was achieved on a CESI 8000 system (AB Sciex, Framingham, MA), coupled via porous sheathless interfacing to an ultra-high resolution (UHR)-qTOF maXis Impact MS system (Bruker Daltonics) operating in positive ion mode. The sample migrated over a 90-cm bare fused capillary (i.d. 30 μm, o.d. 150 μm, AB Sciex). A 10% acetic acid solution was used as background electrolyte and a 50 mm ammonium acetate solution as leading electrolyte. A voltage of 20 kV was applied and 70 nl was injected during 1 min. The ion detection window for both MS1 and MS2 was set at *m/z* 50–2200.

##### Data Processing

The LC-MS(/MS) data was analyzed using DataAnalysis 4.2 software (Bruker Daltonics). Within this program, the function Compounds - AutoMS(n) was used to generate 300 compound spectra (with sum spectra generated for compounds within an *m/z* window of 0.5 Th over a chromatographic peak width of 0.5 min), and these data were deconvoluted and exported as a mascot generic file. Protein identification through primary sequence database searching was performed using the MASCOT search algorithm (MASCOT Daemon version 2.2.2; Matrix Science, London, UK). The following MASCOT settings were used: taxonomy: *Homo sapiens*; database: SwissProt; enzyme: trypsin (for trypsin digests)/no enzyme (for proteinase K digests)/chymotrypsin (for chymotrypsin digests); fixed modifications: carbamidomethyl (C); variable modifications: oxidation (M); max missed cleavages: 1; MS1 peptide tolerance: 0.3 Da; MS/MS tolerance: 0.3 Da; #13C: 0.

The fragmentation spectra were screened manually for common oxonium fragment ions, which are characteristic for glycopeptide fragmentation: *m/z* 366.14, [1 HexNAc (*N*-acetylhexosamine) + 1 Hex (hexose) + H]^+^; *m/z* 292.10, [1 NeuAc (*N*-acetylneuraminic acid) + H]^+^; *m/z* 274.09, [1 NeuAc - H_2_O + H]^+^; *m/z* 657.23, [1 HexNAc + 1 Hex + 1 NeuAc + H]^+^. From the MS/MS spectra of O-glycopeptides, the composition of the glycan and the mass of the peptide moiety could be deduced. This peptide mass was used to generate matching IgG peptide sequences using the ExPASY FindPept software tool (http://www.expasy.org/tools/findpept.html). The MS3 spectrum of the peptide moiety was then used to confirm the peptide sequence, by comparing the peaks in the spectrum to the b and y ions expected according to the Protein Prospector MS-Product tool (http://prospector.ucsf.edu/prospector/cgi-bin/msform.cgi?form=msproduct).

Relative quantification of the O-glycans present on the tryptic IgG3 peptide SCDTPPPCPR was done by summing LC-MS spectra over a fixed time window of 0.5 min surrounding the elution of each (glyco)peptide, and then summing the background-corrected intensities of the first 3 isotopic peaks for each O-glycopeptide. If the compound was present in multiple charge states, the background-corrected peak intensities of the different charge states were summed followed by normalizing the data so that the total was 100%. A separate correction factor was applied to each of the glycopeptides to correct for the fact that higher mass compounds have a lower percentage of signal intensity present in the first 3 isotopic peaks. First, the percentage of signal intensity present in each isotopic peak was calculated using IDCalc version 0.3 (University of Washington). The correction factor consists of the percentage of signal intensity present in the first three isotopic peaks of the peptide divided by the percentage of signal intensity present in the first three isotopic peaks of the O-glycopeptide.

In order to obtain a more accurate determination of the percentage of oligosaccharide occupation of the tryptic IgG3 peptide SCDTPPPCPR, relative quantification was done using LC-MS analysis of a sialidase- and galactosidase-treated tryptic digest sample. The ratio between the peptide and the peptide + HexNAc was determined in the same way as described above.

Relative quantification of N-glycosylation of IgG3 was performed using an adjusted version of the method reported by Selman *et al.* (31). Targeted alignment was done using a novel in-house tool, which aligned nanoLC-ESI-IT-MS spectra according to a list of calibrant *m/z* and retention time values. Various IgG3 N-glycopeptides were used as calibrants. The algorithm examines the *m/z* region within a given time window around each calibrant and isolates the local maxima. The observed retention times of these maxima and the desired retention times are then taken as an input array for a second degree polynomial fit, returning a function which is used to transform the observed retention time of the whole spectrum, resulting in well-aligned spectra. The in-house tool 3D Max Extractor was used to extract signal intensities within specific *m/z* windows. The program examines all data points in *m/z*-retention time space around a given analyte and reports the maximum intensity observed per analyte. Background-corrected intensities of the first three isotopic peaks belonging to IgG2/3 glycopeptides with a charge state of 2+, 3+ or 4+ were determined from the spectra and summed. The glycopeptide values were normalized by dividing by the summed intensity of all glycopeptide values. Glycopeptides were included if they exhibited a signal-to-noise ratio over 3 in at least 25% of the samples. The tryptic peptide that contains the N-glycosylation site has the same mass in both IgG3 and IgG2, and thus with this method, we could not distinguish between IgG3 and IgG2 glycopeptides.

## RESULTS

### 

#### 

##### O-glycosylation of IgG3

Various types of IgG3 samples were obtained: polyclonal IgG3 samples purified from the serum of six donors (44.5 ± 9 years, three male + three female (details in supplemental Table S1)); polyclonal IgG3 purified from pooled plasma; and two recombinant monoclonal anti-GDob1 IgG3 allotypes (G3m(g) and G3m(s)) produced in human embryonic kidney (HEK) cells. Analysis of these samples by SDS-PAGE in reducing condition followed by Coomassie staining revealed an upper band at ∼60 kDa corresponding to the heavy chain of IgG3 and a lower band at ∼30 kDa corresponding to the kappa and lambda light chains (supplemental Fig. S1). The IgG3m(s) sample exhibits a slightly lower mass, which is expected since this IgG3 allotype lacks one of the hinge repeat motifs. The single donor samples were visibly less pure than the other IgG3 samples, which is likely due to differences in sample preparation. The bands were excised and the proteins were reduced using DTT followed by alkylation of cysteine residues and subsequently overnight digestion with either trypsin or proteinase K. The samples were then analyzed by nanoLC-RP-ESI-IT-MS(/MS), and the resulting data were subjected to automated primary sequence database searching for protein identification (supplemental Table S3). All bands at ∼60 kDa were confirmed to contain the IgG3 heavy chain, while the lower bands at 25–30 kDa contained either kappa light chain (in the monoclonal samples) or both kappa and lambda light chains (in the polyclonal samples). The lower band at ∼52 kDa that was visible in the single donor samples (supplemental Fig. S1) was found to contain mainly IgG1, 2, and 3, as determined by examining subclass-specific peptides (data not shown).

**Fig. 1.**
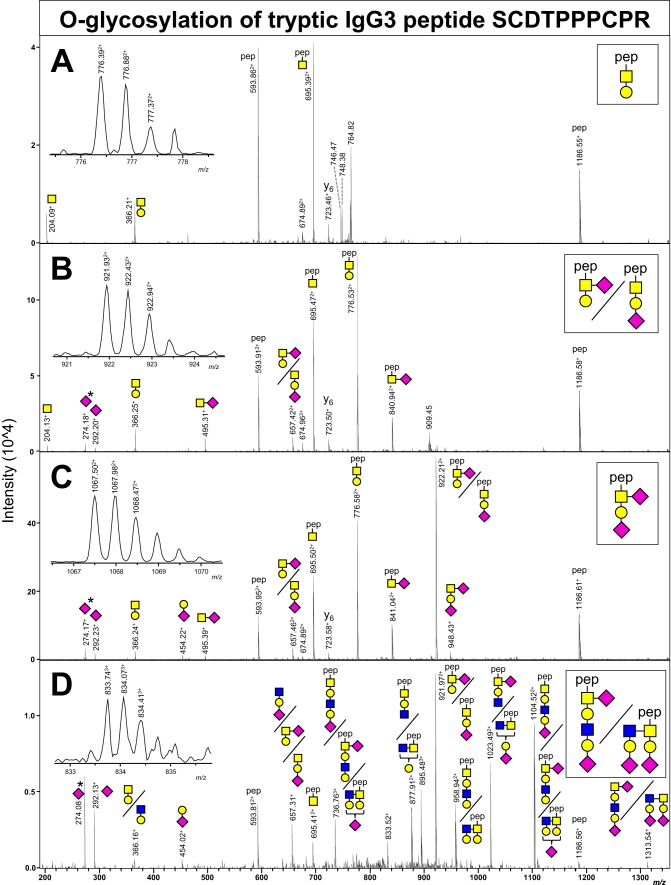
**NanoLC-ESI-IT-CID spectra showing fragmentation of various trypsin-generated recombinant IgG3m(g) O-glycopeptides.** Non-, mono- and disialylated core 1 type O-glycans (*A–C*) were seen attached to the peptide SCDTPPPCPR, as well as a disialylated O-glycan with an *N*-acetyllactosamine (*N*-acetylglucosamine + galactose) (*D*). These structures are partially based on literature since it is difficult to distinguish between different types of hexoses and *N*-acetylhexosamines with mass spectrometry. The triply charged peak at *m/z* 895.48 in panel D has a higher mass than the precursor mass, and thus likely originates from a contaminant. The MS1 precursor peak is shown for each fragmentation spectrum. Pep = peptide; yellow square = *N*-acetylgalactosamine, yellow circle = galactose; blue square = *N*-acetylglucosamine; purple diamond = *N*-acetylneuraminic acid.

O-glycopeptides were detected by searching the LC-MS/MS fragmentation spectra for oxonium fragment ions, which are characteristic for glycopeptide fragmentation: *m/z* 366.14 [HexNAc + Hex + H]^+^; *m/z* 292.10 [NeuAc + H]^+^; *m/z* 274.09 [NeuAc - H_2_O + H]^+^; and *m/z* 657.23 [HexNAc + Hex + NeuAc+ H]^+^. Three types of O-glycans were identified in the trypsin and proteinase K digests of all IgG3 samples: [1 HexNAc + 1 Hex], [1 HexNAc + 1 Hex + 1 NeuAc], and [1 HexNAc + 1 Hex + 2 NeuAc]. The fragmentation spectra of the tryptic glycopeptides are depicted in Fig. 1.

While mass spectrometry does not reveal the exact identity of the monosaccharides, digestion of these glycopeptides by sialidase and galactosidase led to complete trimming of the oligosaccharides (supplemental Fig. S2), thereby confirming the presence of α2-linked *N*-acetylneuraminic acid and β1-linked galactose. Based on literature, we expect the HexNAc attached to the peptide to be an *N*-acetylgalactosamine (32). The encountered glycan structures are therefore presumably a non-, mono-, and disialylated core 1 type O-glycan. A minor fraction but distinct fourth type of O-glycan was seen only in the monoclonal recombinant IgG3 samples: [2 HexNAc + 2 Hex + 2 NeuAc] (Fig. 1*D*). The peak at 1313.54^1+^ in the MS/MS spectrum corresponds to the intact O-glycan B-ion (the nomenclature for glycan fragmentation is described by Domon and Costello (33)), indicating a hexasaccharide O-glycan that may be a core 1 or core 2 type. A comprehensive overview of the *m/z* values in MS1 and MS2 spectra corresponding to the O-glycopeptides can be found in supplemental Table S4.

##### Localization of the IgG3 O-glycosylation Site

**Fig. 2.**
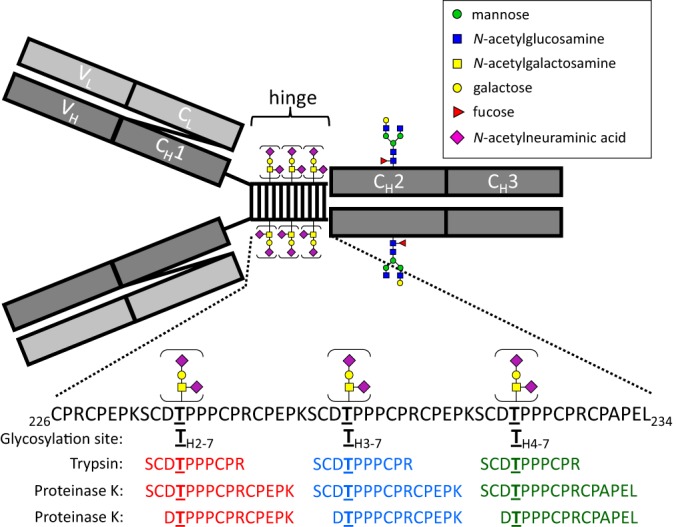
**A schematic overview of IgG3, which consists of two heavy chains (shown in dark gray, one variable and three conserved domains) and two light chains (light gray, one variable and one conserved domain).** Black bars represent interchain disulfide bonds. An N-glycosylation site is present in domain CH2. Each IgG3 heavy chain contains three hinge repeat sequences with two or three partially occupied O-glycosylation sites. The peptide sequences of the major trypsin- and proteinase K-generated O-glycopeptides are shown.

The amino acid sequence of the trypsin- and proteinase K-generated O-glycopeptides was identified through MS3 fragmentation of the peptide. In tryptic digests, the same peptides were also seen without glycans attached, demonstrating that the O-glycosylation sites are only partially occupied. The peptide sequences could be traced back to a triple repeat sequence within the IgG3 hinge region (Fig. 2). This region contains six potential O-glycosylation sites: three threonine residues and three serine residues. The conventional European Union notation for IgG3 (34), which is based on homology between all IgG subclasses, does not cover the triple repeat sequence, and therefore an alternative notation method was introduced to refer to amino acid residues within the IgG3 hinge region. The IgG3 hinge covers four exons, the last three of which encode for the same amino acid sequence (allotype IgG3m(s) forms an exception since it has only two repeats). The location of an amino acid within the hinge region will be specified as “HX-Y,” with “X” standing for the exon number (1/2/3/4) and Y for the amino acid number within the exon (1–17).

The proteinase K-generated glycopeptide _H4–6_DTPPPCPRCPAPEL_234_ demonstrates that T_H4–7_ is occupied, while _H2/3–6_DTPPPCPRCPEPK_H3/4–3_ demonstrates that either T_H2–7_ or T_H3–7_ or both are also occupied. However, the proteinase K digest did not allow us to either confirm or disprove that serine residues were also occupied since all O-glycopeptides that contain a serine residue also contain a threonine residue.

We therefore attempted to use endoproteinase AspN in order to further digest trypsin-generated O-glycopeptides derived from monoclonal IgG3 from “SCDTPPPCPR” to “SC” and “DTPPPCPR.” However, while the majority of the nonglycosylated tryptic hinge repeat peptides were cleaved by AspN, the O-glycopeptides with the same peptide sequence remained undigested. The O-glycopeptides also remained resistant to AspN-digestion after the tryptic O-glycopeptides were treated with sialidase and galactosidase, trimming most of the O-glycans down to a single *N*-acetylhexosamine (data not shown).

**Fig. 3.**
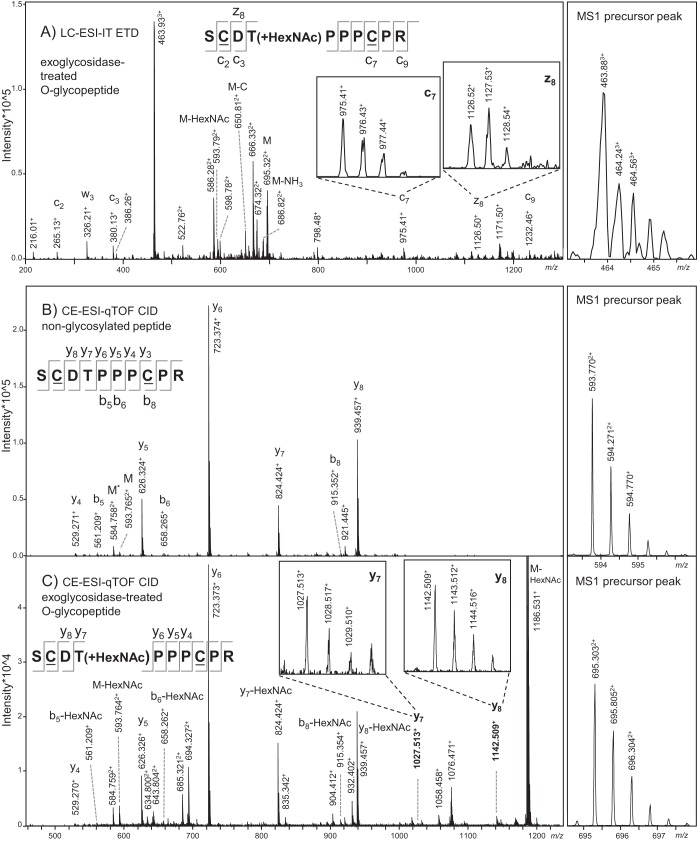
(*A*) NanoLC-ESI-IT-MS/MS with electron transfer dissociation fragmentation of a sialidase- and galactosidase-treated tryptic glycopeptide of recombinant IgG3m(s). Peptide backbone fragmentation (c- and z-ions) and loss of the *N*-acetylhexosamine (HexNAc) or cysteine side chain (*C*) were observed. (*B–C*) CE-ESI-beam-type CID analysis was done on sialidase- and galactosidase-treated tryptic peptides of IgG3 derived from pooled plasma, and fragmentation spectra are shown for (*B*) the unoccupied tryptic hinge peptide SCDTPPPCPR and (*C*) the O-glycosylated version of the same peptide with a single *N*-acetylgalactosamine attached. The b- and y-ions are annotated. The MS1 precursor peak is shown on the right. The doubly charged peak at *m/z* 694.327 in panel C is likely a coeluting compound that appears in the spectrum because it was present within the precursor *m/z* window, and the peak at *m/z* 694.321 is the same compound after loss of water. The cysteine residues have been underlined to denote carbomidomethylation.

In order to obtain further evidence about the position of the O-glycans within the IgG3 hinge region, we performed nanoLC-ESI-IT-MS/MS analysis with electron transfer dissociation on a sialidase- and galactosidase-treated tryptic digest of IgG3m(s). Peptide backbone fragmentation with retention of the glycan moiety could be observed in the resulting mass spectrum, and this appeared to be in line with occupation of the threonine residue (Fig. 3*A*). NanoLC-ESI-IT-MS/MS with CID fragmentation also revealed signals that appear to show peptide backbone fragmentation with glycan retention, but the signal intensity was too low be definitive (data not shown). By using the more sensitive technique t-ITP-CE-ESI-qTOF-MS/MS with CID fragmentation to analyze sialidase- and galactosidase-treated trypsin-digested IgG3 derived from pooled plasma (Figs. 3*B* and 3*C*), we were able to observe minor peaks (y7 and y8) pertaining to peptide backbone fragmentation without glycan loss, indicating occupation of the threonine residue.

To further establish that the O-glycan resides only on the threonine residue, several recombinant anti-TNP IgG3m(g) proteins were produced, in which either the three serines, the three threonines, or all six potential O-glycosylation sites in the hinge repeat region were replaced by alanine, along with wild-type IgG3m(g) as a control (protein sequences are listed in supplemental Table S2). As expected, LC-MS/MS analysis of the wild-type IgG3, digested with trypsin or proteinase K, revealed O-glycosylation of the hinge region, while no O-glycosylation was seen on the IgG3 in which all six potential O-glycosylation sites had been replaced by alanine. IgG3 that only lacked the hinge region serines contained the same O-glycans seen in wild-type IgG3. No O-glycopeptides were found in IgG3 lacking threonines in the hinge repeat, again suggesting that the O-glycans within the IgG3 hinge region reside only on the threonine residue and not on serine (supplemental Fig. S3).

##### Relative Quantification of IgG3 O- and N-glycosylation

**Fig. 4.**
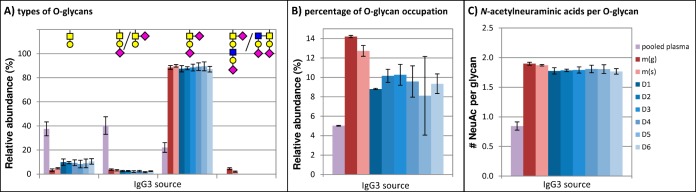
(*A*) Relative quantification of IgG3 O-glycosylation based on nanoLC-ESI-IT-MS analysis of tryptic glycopeptides from various IgG3 samples (IgG3 derived from pooled plasma, two monoclonal IgG3 allotypes and IgG3 purified from six donors (D1–6)). The signal intensities were normalized on the sum of all hinge-derived O-glycopeptides. The relative abundance and technical variation are based on LC-MS analyses of four distinct tryptic digests, each of them measured twice. The values given for glycopeptide NHS are expected to be significantly lower than the actual values because the triply charged compound overlapped with the doubly charged hinge peptide with a putative acetylation modification in all samples, leaving only the doubly charged peak for relative quantification. (*B*) An estimate of the total percentage of O-glycosylation was derived from relative quantification of tryptic (glyco)peptides that had been treated with exoglycosidases, trimming all O-glycans down to a single HexNAc. The averages and standard deviation are based on two LC-MS analyses of the same sample. (*C*) The number of *N*-acetylneuraminic acids per O-glycan was calculated from the same O-glycopeptide data listed under (*A*). A comprehensive list of values is available in supplemental Table S5.

Relative quantification of the O-glycosylation was done using LC-MS data of trypsin-digested IgG3. The abundances of the different glycans attached to the peptide SCDTPPPCPR were normalized to the total of all glycopeptides (Fig. 4*A*). When the O-glycopeptide values were normalized on the sum of both O-glycopeptides and unoccupied peptide, we found the following O-glycopeptide abundances: 3.3% (standard deviation ± 0.6) for IgG3 from pooled plasma; 21.9% (± 2.9) for recombinant IgG3m(g) with three hinge repeats; 18.5% (± 1.8) for IgG3m(s) with two hinge repeats; and 11.6% (± 1.9) on average for single-donor-derived IgG3 (supplemental Table S5). We observed a slight fluctuation in the ratio of the signals of IgG3-hinge-derived O-glycopeptides *versus* unoccupied peptide, which results in a larger technical variation than when we normalize on O-glycopeptides only. It is known that large glycans moieties may result in a considerably lowered ionization of the glycopeptide as compared with the corresponding nonglycosylated peptide (35), making it difficult to compare peptide and glycopeptide signal intensities. Therefore, in an attempt to obtain reliable relative quantification data, tryptic IgG3 O-glycopeptides were treated with exoglycosidases, which should trim all O-glycans down to a single *N*-acetylgalactosamine, with the exception of the minor hexasaccharide glycoform that likely contains an *N*-acetylglucosamine. We observed a low intensity signal at *m/z* 877.85 that appears to be an exoglycosidase digestion product of this hexasaccharide glycan with 2 HexNAc and 1 Hex remaining, indicating that the glycan may be core 1 type. LC-MS analysis of the exoglycosidase-treated tryptic IgG3 (O-glyco)peptides showed that ∼10% of the O-glycosylation sites within IgG3 derived from each of the six donors carried an O-glycan (Fig. 4*B*), and the interindividual differences appear to be relatively small. Monoclonal IgG3 exhibited a slightly higher degree of O-glycosylation, with ∼12–14% of the sites occupied. IgG3 derived from pooled plasma showed a much lower level of O-glycosylation (5%), as well as a markedly lower abundance of the disialylated O-glycan [1 HexNAc + 1 Hex + 2 NeuAc], while showing increased levels of monosialylated O-glycans compared with the other IgG3 samples (Fig. 4*A*). Upon calculation of the number of *N*-acetylneuraminic acids per O-glycan (Fig. 4*C*), the deviation of IgG3 derived from pooled plasma became even more apparent.

In all IgG3 samples, a modified version of the hinge peptide SCDTPPPCPR was observed, with a mass increase of +42.04 Da that could consist of acetylation. This modification was observed on both the nonglycosylated and glycosylated peptide, and the percentage of modified peptide was found to be stable at ∼15% regardless of the type of O-glycan attached or the IgG3 sample source. LC-ESI-IT-MS/MS spectra show that the modification appears to be present on the N-terminal serine or cysteine residue. When IgG3 was digested in-solution without reduction alkylation (data not shown), the modification was not observed on the resulting disulfide bridge-linked hinge peptide dimer, indicating that the modification is an artifact of the sample processing. Furthermore, peaks with masses corresponding to the a hinge peptide with an acrylamide adduct (*m/z* 600.75 (2+)) or with an oxidized disulfide bridge (*m/z* 535.70 (2+)) were also seen, but since their signal intensity was only around 5% or less than 2%, respectively, of the signal intensity of the main peptide peak, we do not expect these modifications to significantly influence our results with regard to glycopeptide quantification.

Because the type and quantity of O-glycosylation appeared similar in IgG3 derived from different single donors, we also investigated interindividual differences in Fc N-glycosylation at position N297. Relative quantification of Fc N-glycosylation was done using tryptic digests of single donor IgG3 (supplemental Fig. S4 and supplemental Table S6). Minor differences were observed between the IgG2/3 N-glycosylation of these donors, most pronounced for the degree of galactosylation.

In-solution trypsin digestion of IgG3 without reduction alkylation produces a dimer of the hinge repeat peptide held together by two interchain disulfide bridges. Mono-glycosylated dimers were observed in all IgG3 samples. In the monoclonal IgG3 samples, a dimer with two disialylated glycans attached was seen at a very low abundance (∼1.0% (G3m(g)) and 0.4% (G3m(s)) of the total of O-glycopeptides + peptide) (supplemental Table S7); in the polyclonal IgG3 samples, we did not observe a di-glycosylated dimer, which may be due to the fact that these samples were less pure than the monoclonal samples and thus had a higher background in LC-MS.

##### O-glycosylation of Fc Constructs with an IgG4 Hinge

Four recombinant IgG Fc constructs produced in HEK cells, all carrying an IgG4 hinge (30), were also found to carry O-glycans (supplemental Fig. S5). The Fc-domains consisted either of the G3m(b) or G3m(c3c5) allotypes for IgG3 or IgG4 with V397M or V397M, and K392N; protein sequences can be reviewed in supplemental Table S2. The O-glycopeptides observed in LC-MS(/MS) analysis of chymotrypsin and proteinase K digests contained one potential glycosylation site: a serine residue in the hinge region at what in intact IgG4 would be position 228; the peptide sequences of the encountered O-glycopeptides are listed in supplemental Table S4.

Because of these findings, we also investigated whether O-glycosylation was present on whole IgG4. We obtained IgG4 from the serum of a patient with high IgG4 levels and from the serum of a rheumatoid arthritis patient, as well as an IgG4 sample produced in HEK cells and a humanized murine IgG4 therapeutic antibody. LC-MS(/MS) analysis of trypsin and proteinase K digests revealed the peptide covering S228, but no O-glycans were observed.

## DISCUSSION

IgG3 stands out from the other IgG subclasses due to its elongated hinge region and enhanced effector functions. In this paper, we demonstrate for the first time partial O-glycosylation of threonine residues within the triple repeat sequence in the hinge region of each IgG3 heavy chain. Non-, mono- and disialylated core 1 type O-glycans were identified in IgG3 from various sources. A hexasaccharide disialylated O-glycan was also present at a very low abundance but only in the monoclonal IgG3 samples.

### 

#### 

##### Relative Abundance and Distribution of IgG3 O-glycosylation

An estimate of the relative abundance of the various O-glycans was determined from nanoLC-ESI-IT-MS analysis of trypsin-generated (glyco)peptides. It should be noted that because the signal of the monosialylated O-glycopeptide in triply charged state overlapped with the doubly charged peak of the hinge repeat peptide with a putative acetylation modification in all samples, only the doubly charged signal of the monosialylated O-glycopeptide was quantified, thereby leading to an underestimation of this O-glycoform. Furthermore, it is known that glycopeptides with different glycan structures can have different response factors (35), and thus the relative abundances we measured may not accurately reflect the real ratios. In order to obtain a more reliable estimate of the percentage of the hinge repeat motif bearing an O-glycan, tryptic IgG peptides were incubated with exoglycosidases, trimming all O-glycans down to a single *N*-acetylhexosamine. A previous study of quantitative measurements of a single glycosylated peptide using nanoLC-ESI-IT-MS found that the glycopeptide with a single *N*-acetylglucosamine produced signal strengths similar to the nonglycosylated peptide (35). Given the structural similarities between *N*-acetylglucosamine and *N*-acetylgalactosamine, we expect our method of relative quantification of desialylated and degalactosylated O-glycopeptides to give a reliable estimate of the degree of O-glycosylation.

Approximately 10% of the hinge repeat threonines carry an O-glycan in polyclonal IgG3 derived from donor sera. Despite differences in the age and sex of the donors, the degree and type of O-glycosylation appears similar in all six samples. This is in contrast to the N-glycosylation profiles of these samples, which do show interindividual differences in agreement with the literature (36). In the two monoclonal IgG3 allotypes, we examined, IgG3m(g) and IgG3m(s), ∼12–14% of the O-glycosylation sites were occupied; this increase may be due to the expression system (HEK cells) in which these antibodies were produced (37).

A clear difference in O-glycosylation was seen between the commercially obtained IgG3 sample derived from pooled plasma and the other IgG3 samples: The former exhibited a much lower level of terminal *N*-acetylneuraminic acids per O-glycan, as well as a lower level of O-glycosylation overall. Two explanations could be found in the details of the preparation process that were supplied by the manufacturer of this IgG3 sample: Boric acid was used for precipitation and a jacalin column was used to remove a minor IgA contaminant. Acidic treatment has been known to cause loss of terminal N-acetylneuraminic acid (38). However, the N-glycans of pooled-plasma-derived IgG3 did not show a decrease in sialylation compared with single-donor-derived IgG3, which makes it unlikely that boric acid treatment is responsible for the lower level of O-glycan sialylation. Jacalin is a plant lectin with affinity for core 1 type O-glycans; it binds O-glycosylated proteins such as IgA1, as well as several animal IgGs that are suspected of carrying O-glycosylation (39, 40). However, it has been reported that jacalin has a higher affinity for nonsialylated than for sialylated O-glycans in the case of fetuin (40), and the same was observed for monoclonal IgG3 that was run over a jacalin column and found to be depleted for nonsialylated and partially depleted for monosialylated but not for disialylated O-glycans (unpublished data). This does not explain why the pooled plasma-derived IgG3 would exhibit lower levels of disialylated O-glycans and higher levels of monosialylated O-glycans compared with single donor serum-derived IgG3. In any case, it is difficult to say anything about possible depletion without being able to observe the O-glycosylation profile of the original non-jacalin-treated pooled plasma sample. Because it was shown that jacalin interacts with the O-glycosylated IgG3 hinge and because of the deviating O-glycosylation profile obtained from the jacalin-treated pooled plasma-derived sample *versus* IgG3 from single human donors obtained without jacalin depletion, the results obtained for this IgG3 sample will not be deemed representative for the overall human population.

It is likely that the O-glycosylation is distributed randomly among all IgG3s, since monoclonal cell cultures were found to produce partially O-glycosylated instead of either fully or non-O-glycosylated IgG3. Furthermore, hinge peptide-dimer repeats linked by interchain disulfide bridges rarely carry two O-glycans, which indicates that the O-glycosylation is not clustered on a subset of the IgG3 molecules. If the O-glycosylation is distributed randomly, approximately half (1 − probability of 6 unoccupied sites = 1 − ((1 − 0.10)^6) = 47%) of the IgG3s would be expected to carry at least one O-glycan. We observed that less than 1% of hinge peptide-dimer repeats carried two disialylated O-glycans in monoclonal IgG3 samples, whereas the expected value assuming random O-glycosylation site occupancy would be ∼3.8% (G3m(g)) and 2.8% (G3m(s)). These findings indicate that addition of O-linked glycans is inhibited if a glycan is already present at the adjacent site on the opposing heavy chain, thus dispersing the O-glycosylation across a higher percentage of IgG3s.

##### Criteria Determining IgG Hinge-Region O-glycosylation

Based on the amino acid sequence of the IgG3 hinge repeat region, it is not surprising that it forms a target for O-glycosylation. It is known that O-glycosylation occurs in sequences with a high abundance of proline residues, preferentially at position −1 and +3 relative to the glycosylation site (41). In the IgG3 hinge repeat, six of the 16 amino acid residues consist of prolines, including one at position +3 relative to the glycosylation site. Furthermore, due to its extended formation, the IgG3 hinge region has a high degree of surface accessibility (42), which is also associated with O-glycosylation (43). Surface accessibility may be responsible for the slightly lower degree of O-glycosylation observed in recombinant IgG3m(s) compared with m(g): the shorter length of the hinge region of IgG3m(s), which contains two hinge repeats instead of three, could decrease the accessibility for glycosyltransferases. No O-glycosylation was observed at the threonine residues at the N-terminal end of the hinge, T_H1–4_,T_H1–9_ and T_H1–10_, which may be due to either the local amino acid sequence or the secondary structure of this region.

O-glycosylation was also found to be present on the IgG4 hinge of Fc constructs with IgG4 or IgG3 CH2 and CH3 domains. This is not surprising considering the high abundance of proline residues (at position −4, −3, −1, 2, and 4 relative to O-glycosylation site S228) in the IgG4 hinge region, which are a well-known marker for O-glycosylation (41). However, intact IgG4, whether purified from serum or expressed in the same HEK cell expression system as the Fc constructs, was not found to carry hinge region O-glycosylation. It is therefore likely that the presence of the Fab fragment influences the accessibility of the hinge region, preventing the attachment of an O-glycan in the Golgi apparatus in the case of intact IgG4. Similarly, O-glycosylation was recently reported on an IgG1 Fc-fragment produced in HEK and CHO cells but not on intact IgG1 produced under the same circumstances (44). This reinforces the notion that accessibility is essential for the attachment of O-glycans (43). For human IgG3, this is provided by the extensive length of its IgG3 hinge, which in the intact IgG molecules for other IgG subclasses is shielded by the Fab fragments.

##### Function of IgG3 O-glycosylation

The function of the O-glycans found on the IgG3 hinge region has not been investigated, but previous findings give rise to several options. First, the hinge glycosylation might prohibit proteolytic degradation. The IgG hinge is the target of a number of bacterial or endogenous proteases (45, 46), and the extended structure of the IgG3 hinge is presumably the reason that IgG3 is more susceptible to proteolytic degradation than the other IgG subclasses (47–49). We found that tryptic IgG3 O-glycopeptides were resistant against endoprotease AspN digestion, while the corresponding nonglycosylated peptides were not, thus providing evidence that the hinge region O-glycans can shield the hinge region from proteolytic cleavage. In contrast, we did not observe any evidence of incomplete trypsin digestion in the form of miscleaved O-glycopeptides. O-glycosylation has also been found to mediate protease resistance in other immunoglobulins, such as mouse IgG2b and IgA (23, 27). Another function for the hinge region oligosaccharides could consist of helping the hinge region maintain an extended conformation, which might contribute to the flexibility and orientation of the Fab fragments and thereby influence divalent binding to target antigens.

Furthermore, it could be speculated that deviations in the O-glycosylation of IgG3 may have pathological effects, as has been described for IgA nephropathy (50). Aberrant glycosylation of IgA1 is known to play a causal role in IgA nephropathy: The truncated O-glycans are recognized by immunoglobulins, leading to the formation of antibody complexes that are deposited on blood vessel walls near the glomerulus (50, 51). A similar mechanism involving IgG3 O-glycosylation could be theorized to play a role in IgG nephropathy. It has been shown that patients with membranoproliferative glomerulonephritis, a subtype of primary glomerulopathy, have IgG deposits that consist predominantly of IgG3, which may be in line with a causal role for IgG3 O-glycosylation (52). Further research is required to determine the exact nature and functional consequences of IgG3 hinge glycosylation.

## Supplementary Material

Supplemental Data

## Footnotes

^1^ The abbreviations used are:

ABC: ammonium bicarbonate
C1q: complement component 1 q
CE: capillary electrophoresis
CH: conserved heavy chain
CID: collision-induced dissociation
Fab: fragment antigen-binding
Fc: fragment crystallizable
FcγR: Fc-gamma receptor
GDob1: chimeric MN12H2 antibodies with V genes from the human monoclonal IgG2 antibody DOB1
HEK: human embryonic kidney
Hex: hexose
HexNAc: *N*-acetylhexosamine
IgG: immunoglobulin gamma
IT: ion trap
NeuAc: *N*-acetylneuraminic acid
t-ITP: transient isotachophoresis
TNP: trinitrophenol.
