# Supplementary material for: Hinge-Region O-Glycosylation of Human Immunoglobulin G3 (IgG3)
Source: Mol Cell Proteomics. 2015 Mar 10;14(5):1373–84. doi: 10.1074/mcp.M114.047381 (PMC4424406; doi:10.1074/mcp.M114.047381)
Supplement: Supplemental Data [file supp_14_5_1373__index.html]

Hinge-region O-glycosylation of human immunoglobulin G3 (IgG3) — Hinge-Region O-Glycosylation of Human Immunoglobulin G3 (IgG3) — Hinge-Region O-Glycosylation of Human IgG3 — Supplemental Data 

# Hinge-Region O-Glycosylation of Human Immunoglobulin G3 (IgG3)

## Supplemental Data

**Files in this Data Supplement:**

- Supplemental information - Supplemental information
- Supplemental Table S4 - Supplemental Table S4
