# Supplementary material for: Hinge-Region O-Glycosylation of Human Immunoglobulin G3 (IgG3)
Source: Mol Cell Proteomics. 2015 Mar 10;14(5):1373–84. doi: 10.1074/mcp.M114.047381 (PMC4424406; doi:10.1074/mcp.M114.047381)
Supplement: Supplemental Data [file supp_M114.047381_mcp.M114.047381-1.pdf]

Hinge-region O-glycosylation of human immunoglobulin G3 (IgG3)

Supplemental information

Rosina Plomp[1], Gillian Dekkers[2], Yoann Rombouts[1,3], Remco Visser[2], Carolien A. M. Koeleman[1], Guinevere S. M. Kammeijer[1], Bas C. Jansen[1], Theo Rispens[4], Paul J. Hensbergen[1], Gestur Vidarsson[2], Manfred Wuhrer[1,5,*]

1 Center for Proteomics and Metabolomics, Leiden University Medical Center, Leiden, The Netherlands

2 Department of Experimental Immunohematology, Sanquin Research, and Landsteiner Laboratory, Academic Medical Center, University of Amsterdam, Amsterdam, The Netherlands

3 Department of Rheumatology, Leiden University Medical Center, Leiden, The Netherlands

4 Department of Immunopathology, Sanquin Research, and Landsteiner Laboratory, Academic Medical Center, University of Amsterdam, Amsterdam, The Netherlands

5 Division of BioAnalytical Chemistry, VU University Amsterdam, Amsterdam, The Netherlands

Supplemental Table S1. The age and sex of the IgG3 serum donors. M = male, F = female.

Donor	Age (years)	Sex
**D1**	45	F
**D2**	56	M
**D3**	32	M
**D4**	40	F
**D5**	52	M
**D6**	42	F
**average**	44.5	-
**standard deviation**	8.6	-

Supplemental Table S2. The protein sequences of the IgG3 monoclonals and IgG3/4 Fc constructs used in this study are shown below. Potential O-glycosylation sites within the hinge region are colored red. IgG3 amino acid substitutions that differ from the Uniprot IgG3 sequence are highlighted in blue.

IgG3/4 sample	sample information	protein sequence	Potential O-glycosylation sites within the hinge region*
Uniprot sequence of the conserved part of IgHG3 (allotype G3m(b))		ASTKGPSVFPLAPCSRSTSGGTAALGCLVKDYFPEPVTVSWNS<br>GALTSGVHTFPAVLQSSGLYSLSSVVTVPSSSLGTQTYTCNVNH<br>KPSNTKVDKRVELKTPLGDTTHTCPRCPEPKSCDTPPPCPRCPE<br>PKSCDTPPPCPRCPEPKSCDTPPPCPRCPAPELLGGPSVFLFPP<br>KPKDTLMISRTPEVTCVVVDVSHEDPEVQFKWYVDGVEVHNAK<br>TKPREEQYNSTFRVVSVLTVLHQDWLNGKEYKCKVSNKALPAPI<br>EKTISKTKGQPREPQVYTLPPSREEMTKNQVSLTCLVKGFYPSDI<br>AVEWESSGQPENNYNTTPPMLDSDGSFFLYSKLTVDKSRWQQ<br>GNIFSCSVMHEALHNRFTQKSLSLSPGK	T_{H2-7}, T_{H3-7}, T_{H4-7}
IgG3m(g)	anti-Gdob1 IgG3m(g)	MACPGFLWALVISTCLEFSMAEVQLVESGGGLVTFGGSLTLSCA<br>ASGFTFSRAWLTWVRQAPGGGLEWVGRILRMADGGATDYAAT<br>VKGRFTISRDDSKNTVYLHMNNLKTEDTAVYYCANENFWRLDN<br>WGQGTLVTVSSASTKGPSVFPLAPCSRSTSGGTAALGCLVKDY<br>FPEPVTVSWNSGALTSGVHTFPAVLQSSGLYSLSSVVTVPSSSL<br>GTQTYTCNVNHKPSNTKVDKRVELKTPLGDTTHTCPRCPEPKS<br>CDTPPPCPRCPEPKSCDTPPPCPRCPEPKSCDTPPPCPRCPAP<br>ELLGGPSVFLFPPKPKDTLMISRTPEVTCVVVDVSHEDPEVQFK<br>WYVDGVEVHNAKTKLREEQYNSTFRVVSVLTVLHQDWLNGKEY<br>KCKVSNKALPAPIEKTISKAKGQPREPQVYTLPPSREEMTKNQV<br>SLTCLVKGFYPSDIAVEWESNGQPENNYNTTPPMLDSDGSFFLY<br>SKLTVDKSRWQQGNIFSCSVMHEALHNRYTQKSLSLSPGK	T_{H2-7}, T_{H3-7}, T_{H4-7}
IgG3m(s)	anti-Gdob1 IgG3m(s)	MACPGFLWALVISTCLEFSMAEVQLVESGGGLVTFGGSLTLSCA<br>ASGFTFSRAWLTWVRQAPGGGLEWVGRILRMADGGATDYAAT<br>VKGRFTISRDDSKNTVYLHMNNLKTEDTAVYYCANENFWRLDN<br>WGQGTLVTVSSASTKGPSVFPLAPCSRSTSGGTAALGCLVKDY<br>FPEPVTVSWNSGALTSGVHTFPAVLQSSGLYSLSSVVTVPSSNF<br>GTQTYTCNVNHKPSNTKVDKRVELKTPLGDTTHTCPRCPEPKS<br>CDTPPPCPRCPEPKSCDTPPPCPRC*PAPELLGGPSVFLFPPKP<br>KDTLMISRTPEVTCVVVDVSHEDPEVQFKWYVDGVEVHNAKTK<br>PREEQYNSTFRVVSVLTVLHQDWLNGKEYKCKVSNKALPAPIEK<br>TISKTKGQPREPQVYTLPPSREEMTKNQVSLTCLVKGFYPSDIA<br>MEWESSGQPENNYKTTPPVLDSDGSFFLYSKLTVDKSRWQQG<br>NIFSCSVMHEALHNHYTQKSLSLSPGK	T_{H2-7}, T_{H3-7}
IgG3m(g) hinge mutant 3S+3T ➔ 6A	Anti-TNP IgG3m(g)	MACPGFLWALVISTCLEFSMAEVQLVESGGGLVTFGGSLTLSCA<br>ASGFTFSRAWLTWVRQAPGGGLEWVGRILRMADGGATDYAAT<br>VKGRFTISRDDSKNTVYLHMNNLKTEDTAVYYCANENFWRLDN<br>WGQGTLVTVSSASTKGPSVFPLAPCSRSTSGGTAALGCLVKDY<br>FPEPVTVSWNSGALTSGVHTFPAVLQSSGLYSLSSVVTVPSSSL<br>GTQTYTCNVNHKPSNTKVDKRVELKTPLGDTTHTCPRCPEPKA<br>CDAPPPCPRCPEPKACDAPPPCPRCPEPKACDAPPPCPRCPA<br>PELLGGPSVFLFPPKPKDTLMISRTPEVTCVVVDVSHEDPEVQF<br>KWYVDGVEVHNAKTKLREEQYNSTFRVVSVLTVLHQDWLNGKE<br>YKCKVSNKALPAPIEKTISKAKGQPREPQVYTLPPSREEMTKNQ<br>VSLTCLVKGFYPSDIAVEWESNGQPENNYNTTPPMLDSDGSFFL<br>YSKLTVDKSRWQQGNIFSCSVMHEALHNRYTQKSLSLSPGK	-
IgG3m(g) hinge mutant 3T➔3A	Anti-TNP IgG3m(g)	MACPGFLWALVISTCLEFSMAEVQLVESGGGLVTFGGSLTLSCA<br>ASGFTFSRAWLTWVRQAPGGGLEWVGRILRMADGGATDYAAT<br>VKGRFTISRDDSKNTVYLHMNNLKTEDTAVYYCANENFWRLDN<br>WGQGTLVTVSSASTKGPSVFPLAPCSRSTSGGTAALGCLVKDY<br>FPEPVTVSWNSGALTSGVHTFPAVLQSSGLYSLSSVVTVPSSSL<br>GTQTYTCNVNHKPSNTKVDKRVELKTPLGDTTHTCPRCPEPKS<br>CDAPPPCPRCPEPKSCDAPPPCPRCPEPKSCDAPPPCPRCPA<br>PELLGGPSVFLFPPKPKDTLMISRTPEVTCVVVDVSHEDPEVQF<br>KWYVDGVEVHNAKTKLREEQYNSTFRVVSVLTVLHQDWLNGKE<br>YKCKVSNKALPAPIEKTISKAKGQPREPQVYTLPPSREEMTKNQ<br>VSLTCLVKGFYPSDIAVEWESNGQPENNYNTTPPMLDSDGSFFL<br>YSKLTVDKSRWQQGNIFSCSVMHEALHNRYTQKSLSLSPGK	-

IgG3m(g) hinge mutant 3S→3A	Anti-TNP IgG3m(g)	MACPGFLWALVISTCLEFSMAEVQLVESGGGLVTFGGSLTLSCA<br>ASGFTFSRAWLTWVRQAPGGGLEWVGRILRMADGGATDYAAT<br>VKGRFTISRDDSKNTVYLHMNNLKTEDTAVYYCANENFWRLDN<br>WGQGTLVTVSSASTKGPSVFPLAPCSRSTSGGTAALGCLVKDY<br>FPEPVTVSWNSGALTSGVHTFPAVLQSSGLYSLSSVVTVPSSSL<br>GTQTYTCNVNHKPSNTKVDKRVELKTPLGDTTHTCPRCPEPKA<br>CDTPPPCPRCPEPKACDTPPPCPRCPEPKACDTPPPCPRCPAP<br>ELLGGPSVFLFPPKPKDTLMISRTPEVTCVVVDVSHEDPEVQFK<br>WYVDGVEVHNAKTKLREEQYNSTFRVVSVLTVLHQDWLNGKEY<br>KCKVSNKALPAPIEKTISKAKGQPREPQVYTLPPSREEMTKNQV<br>SLTCLVKGFYPSDIAVEWESNGQPENNYNTTPPMLDSDGSFFLY<br>SKLTVDKSRWQQGNIFSCSVMHEALHNRYTQKSLSLSPGK	T_{H2-7}, T_{H3-7}, T_{H4-7}
IgG3m(b)-Fc-h4	IgG3 Fc construct consisting of IgG4 hinge + IgG3m(b) domains CH2&3	ESKYGPPCPSCPAPELLGGPSVFLFPPKPKDTLMISRTPEVTCV<br>VVDVSHEDPEVQFKWYVDGVEVHNAKTKPREEQYNSTFRVVSV<br>LTVLHQDWLNGKEYKCKVSNKALPAPIEKTISKTKGQPREPQVY<br>TLPPSREEMTKNQVSLTCLVKGFYPSDIAVEWESSGQPENNYNT<br>TPPMLDSDGSFFLYSKLTVDKSRWQQGNIFSCSVMHEALHNRF<br>TQKSLSLSPGK	S_{228}
IgG3m(c3c5)-Fc-h4	IgG3 Fc construct consisting of IgG4 hinge + IgG3m(c3c5) domains CH2&3	ESKYGPPCPSCPAPELLGGPSVFLFPPKPKDTLMISRTPEVTCV<br>VVDVSHEDPEVQFKWYVDGVEVHNAKTKPREEQYNSTFRVVSV<br>LTVLHQDWLNGKEYKCKVSNKALPAPIEKTISKTKGQPREPQVY<br>TLPPSREEMTKNQVSLTCLVKGFYPSDIAVEWESSGQPENNYNT<br>TPPVLDSDGSFFLYSRLTVDKSRWQEGNVFSCSVMHEALHNRF<br>TQKSLSLSPGK	S_{228}
IgG4-Fc-V397M	recombinant IgG4 Fc construct consisting of IgG4 hinge + IgG4 domains CH2&3	ESKYGPPCPSCPAPEFLGGPSVFLFPPKPKDTLMISRTPEVTCV<br>VVDVSQEDPEVQFNWYVDGVEVHNAKTKPREEQFNSTYRVVS<br>VLTVLHQDWLNGKEYKCKVSNKGLPSSIEKTISKAKGQPREPQV<br>YTLPPSQEEMTKNQVSLTCLVKGFYPSDIAVEWESNGQPENNY<br>KTTPPMLDSDGSFFLYSRLTVDKSRWQEGNVFSCSVMHEALHN<br>HYTQKSLSLSLGK	S_{228}
IgG4-Fc-V397M,K392N	recombinant IgG4 Fc construct consisting of IgG4 hinge + IgG4 domains CH2&3	ESKYGPPCPSCPAPEFLGGPSVFLFPPKPKDTLMISRTPEVTCV<br>VVDVSQEDPEVQFNWYVDGVEVHNAKTKPREEQFNSTYRVVS<br>VLTVLHQDWLNGKEYKCKVSNKGLPSSIEKTISKAKGQPREPQV<br>YTLPPSQEEMTKNQVSLTCLVKGFYPSDIAVEWESNGQPENNY<br>NTTPPMLDSDGSFFLYSRLTVDKSRWQEGNVFSCSVMHEALHN<br>HYTQKSLSLSLGK	S_{228}

* hinge region notation: for the IgG3 hinge region notation, H# stands for the number of the hinge region exon, while the second number pertains to the amino acid sequence within the exon. The notation according to the EU convention is used for the IgG4 hinge region. An overview of this notation can be found at http://www.imgt.org/IMGTScientificChart/Numbering/Hu_IGHGnber.html.

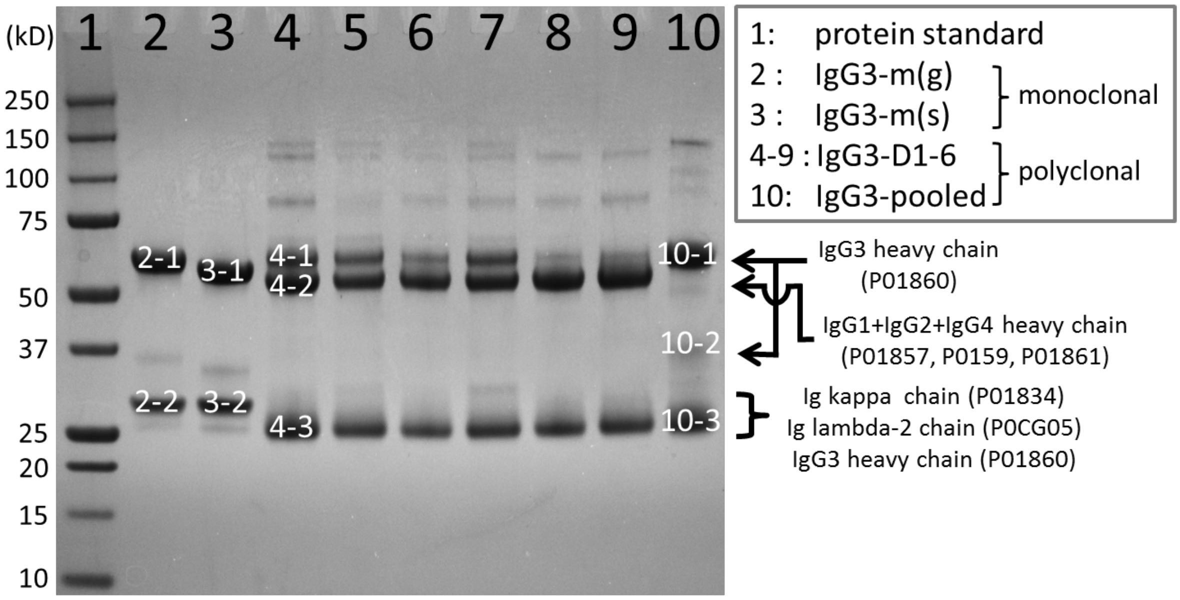

Supplemental Figure S1. SDS-PAGE analysis of various IgG3 samples. The bands denoted with a numerical code were excised and the embedded proteins were digested with trypsin, followed by LC-ESI-IT-MS(/MS) analysis. Automated primary sequence database searching using MASCOT was used to identify the IgG3 heavy chain, as well as both kappa and lambda (in polyclonal samples) or only kappa (in monoclonal samples) light chains, which are listed here with their primary UniProt accession number. A more comprehensive list of the identified proteins can be seen in Supplemental Table S3.

Supplemental Table S3. An overview of the proteins identified from nanoLC-ESI-IT-MS/MS of trypsin-generated IgG3 glycopeptides. The IgG3 was run on an SDS PAGE gel (Suppl. Fig. S1), the annotated bands were excised and the embedded proteins were digested with trypsin and analysed with LC-ESI-MS(/MS) with CID fragmentation. Automated primary sequence database searching (MASCOT) was used to identify the peptides present in each of the bands. Proteins were included in this overview if at least two unique peptides with a MASCOT score of 30 or higher were found. The settings used for MASCOT are listed in the 'Experimental procedures' section of the paper.

**2-1: IgG3m(g)**			
**Protein Name**	**Peptide Sequence**	**Modifications**	**Highest Peptide Score**
Ig gamma-3 chain C region, P01860	VVSVLTVLHQDWLNGK		31
	TPEVTCVVVDVSHEDPEVQFK		81
	TPLGDTTHTCPR		81
	WYVDGVEVHNAK		84
	WQQGNIFSCSVMHEALHNR		83
	VELKTPLGDTTHTCPR		61
	STSGGTAALGCLVK		112
	NQVSLTCLVK		66
	EPQVYTLPPSREEMTK		46
	GPSVFPLAPCSR		61
	SCDTPPPCPR		53
	CPAPELLGGPSVFLFPPKPK		46

**3-1: IgG3m(s)**			
**Protein Name**	**Peptide Sequence**	**Modifications**	**Highest Peptide Score**
Ig gamma-3 chain C region, P01860	VVSVLTVLHQDWLNGK		30
	TPLGDTTHTCPR		85
	WYVDGVEVHNAK		55
	VELKTPLGDTTHTCPR		55
	STSGGTAALGCLVK		93
	NQVSLTCLVK		63
	GQPREPQVYTLPPSREEMTK		39
	DTLMISR		44
	EPQVYTLPPSREEMTK		41
	GPSVFPLAPCSR		53
	SCDTPPPCPR		57
	EPQVYTLPPSREEM*TK	Oxidation (M)	42
Ig gamma-1 chain C region, P01857	VVSVLTVLHQDWLNGK		30
	TTPPVLDSDGSFFLYSK		83
	DTLMISR		44
	STSGGTAALGCLVK		93
	NQVSLTCLVK		63

**4-1: IgG3 from single donor serum**			
**Protein Name**	**Peptide Sequence**	**Modifications**	**Highest Peptide Score**
Ig gamma-3 chain C region, P01860	TPLGDTTHTCPR		45
	WQQGNIFSCSVMHEALHNR		31
	STSGGTAALGCLVK		84
	DTLM*ISR	Oxidation (M)	40
	NQVSLTCLVK		46
	DTLMISR		43
	EPQVYTLPPSR		46
	GPSVFPLAPCSR		56
	SCDTPPPCPR		53
	WQQGNIFSCSVM*HEALHNR	Oxidation (M)	56
Ig gamma-1 chain C region, P01857	TPEVTCVVVDVSHEDPEVK		51
	EPQVYTLPPSR		46
	DTLMISR		43
	GPSVFPLAPSSK		65
	STSGGTAALGCLVK		84
	DTLM*ISR	Oxidation (M)	40
	NQVSLTCLVK		46
Serum albumin, P02768	TCVADESAENCDK		78
	RPCFSALEVDETYVPK		37
	YICENQDSISSK		45

**4-2: IgG3 from single donor serum**			
**Protein Name**	**Peptide Sequence**	**Modifications**	**Highest Peptide Score**
Ig gamma-3 chain C region, P01860	TPLGDTTHTCPR		48
	STSGGTAALGCLVK		83
	DTLM*ISR	Oxidation (M)	42
	NQVSLTCLVK		50
	DTLMISR		43
	EPQVYTLPPSR		46
	GPSVFPLAPCSR		51
	ALPAPIEK		30
	SCDTPPPCPR		55
Ig gamma-1 chain C region, P01857	TPEVTCVVVDVSHEDPEVK		52
	GPSVFPLAPSSK		56
	STSGGTAALGCLVK		83
	DTLM*ISR	Oxidation (M)	42
	NQVSLTCLVK		50
	DTLMISR		43
	EPQVYTLPPSR		46
	ALPAPIEK		30
Ig gamma-2 chain C region, P01859	EPQVYTLPPSR		46
	DTLMISR		43
	GPSVFPLAPCSR		51
	STSESTAALGCLVK		68
	DTLM*ISR	Oxidation (M)	42
	NQVSLTCLVK		50
Ig gamma-4 chain C	DTLMISR		43

region, P01861	GPSVFPLAPCSR		51
	STSESTAALGCLVK		68
	DTLM*ISR	Oxidation (M)	42
	NQVSLTCLVK		50
	EPQVYTLPPSQEEM*TK	Oxidation (M)	34
Keratin, type I cytoskeletal 9, P35527	FSSSSGYGGGSSR		99
	GGSGGSYGGGGSGGGYGGGSGSR		88

**10-1: IgG3 from pooled plasma**			
**Protein Name**	**Peptide Sequence**	**Modifications**	**Highest Peptide Score**
Ig gamma-3 chain C region, P01860	TPEVTCVVVDVSHEDPEVQFK		60
	TPLGDTTHTCPR		66
	WYVDGVEVHNAK		83
	WQQGNIFSCSVMHEALHNR		64
	STSGGTAALGCLVK		108
	NQVSLTCLVK		69
	EPQVYTLPPSREEMTK		34
	GPSVFPLAPCSR		56
	SCDTPPPCPR		56
	CPAPELLGGPSVFLFPPKPK		50
	EPQVYTLPPSREEM*TK	Oxidation (M)	35
Ig heavy chain V-III region BRO, P01766	AEDTAVYYCAR		83
	EVQLVESGGGLVQPGGSLR		99

**2-2: IgG3m(g)**			
**Protein Name**	**Peptide Sequence**	**Modifications**	**Highest Peptide Score**
Ig gamma-3 chain C region, P01860	GPSVFPLAPCSR		41
	TPLGDTTHTCPR		34
	SCDTPPPCPR		50
	WYVDGVEVHNAK		36
	STSGGTAALGCLVK		42

**3-2: IgG3m(s)**			
**Protein Name**	**Peptide Sequence**	**Modifications**	**Highest Peptide Score**
Ig kappa chain C region, P01834	VDNALQSGNSQESVTEQDSK		67
	TVAAPSVFIFPPSDEQLK		58
	VYACEVTHQGLSSPVTK		76
	HKVYACEVTHQGLSSPVTK		67
Ig gamma-3 chain C region, P01860	TPLGDTTHTCPR		32
	WYVDGVEVHNAK		36
	DTLM*ISR	Oxidation (M)	40
Ig gamma-1 chain C region, P01857	TTPPVLDSDGSFFLYSK		35
	DTLM*ISR	Oxidation (M)	40

**4-3: IgG3 from single donor serum**			
**Protein Name**	**Peptide Sequence**	**Modifications**	**Highest Peptide Score**
Ig gamma-3 chain C region, P01860	EPQVYTLPPSR		37
Ig gamma-3 chain C region, P01860	DTLMISR		43
Ig gamma-3 chain C region, P01860	GPSVFPLAPCSR		52
Ig gamma-3 chain C region, P01860	TPLGDTTHTCPR		40
Ig gamma-3 chain C region, P01860	SCDTPPPCPR		39
Ig gamma-3 chain C region, P01860	ALPAPIEK		30
Ig gamma-3 chain C region, P01860	DTLM*ISR	Oxidation (M)	40
Ig gamma-3 chain C region, P01860	NQVSLTCLVK		43
Ig gamma-1 chain C region, P01857	EPQVYTLPPSR		37
Ig gamma-1 chain C region, P01857	DTLMISR		43
Ig gamma-1 chain C region, P01857	GPSVFPLAPSSK		39
Ig gamma-1 chain C region, P01857	ALPAPIEK		30
Ig gamma-1 chain C region, P01857	DTLM*ISR	Oxidation (M)	40
Ig gamma-1 chain C region, P01857	NQVSLTCLVK		43
Ig kappa chain V-I region EU, P01598	ASSLESGVPSR		79
Ig kappa chain V-I region EU, P01598	DIQMTQSPSTLSASVGDR		120
Ig kappa chain V-I region EU, P01598	DIQM*TQSPSTLSASVGDR	Oxidation (M)	91
Ig kappa chain C region, P01834	VDNALQSGNSQESVTEQDSK		93
Ig kappa chain C region, P01834	VYACEVTHQGLSSPVTK		65
Ig kappa chain V-III region SIE, P01620	LLIYGASSR		41
Ig kappa chain V-III region SIE, P01620	FSGSGSGTDFTLTISR		95
Ig kappa chain V-I region AU, P01594	DIQM*TQSPSSLSASVGDR	Oxidation (M)	77
Ig kappa chain V-I region AU, P01594	DIQMTQSPSSLSASVGDR		92
Immunoglobulin lambda-like polypeptide 5, B9A064	SYSCQVTHEGSTVEK		39
Immunoglobulin lambda-like polypeptide 5, B9A064	VTVLGQPK		35

**10-2: IgG3 from pooled plasma**			
**Protein Name**	**Peptide Sequence**	**Modifications**	**Highest Peptide Score**
Ig gamma-3 chain C region, P01860	TPEVTCVVVDVSHEDPEVQFK		65
Ig gamma-3 chain C region, P01860	WYVDGVEVHNAK		79
Ig gamma-3 chain C region, P01860	STSGGTAALGCLVK		87
Ig gamma-3 chain C region, P01860	NQVSLTCLVK		77
Ig gamma-3 chain C region, P01860	EPQVYTLPPSR		32
Ig gamma-3 chain C region, P01860	EPQVYTLPPSREEMTK		44
Ig gamma-3 chain C region, P01860	GPSVFPLAPCSR		69
Ig gamma-3 chain C region, P01860	SCDTPPPCPR		54

**10-3: IgG3 from pooled plasma**			
**Protein Name**	**Peptide Sequence**	**Modifications**	**Highest Peptide Score**
Ig kappa chain C region, P01834	VDNALQSGNSQESVTEQDSK		87
Ig kappa chain C region, P01834	TVAAPSVFIFPPSDEQLK		57
Ig kappa chain C region, P01834	ADYEKHKVYACEVTHQGLSSPVTK		33
Ig kappa chain C region, P01834	VYACEVTHQGLSSPVTK		66
Ig kappa chain C region, P01834	VQWKVDNALQSGNSQESVTEQDSK		52

Ig lambda-2 chain C regions, P0CG05	SYSCQVTHEGSTVEK		60
	SYSCQVTHEGSTVEKTVAPTECS		50
	YAASSYLSLTPEQWK		38
	ADSSPVKAGVETTTPSK		32
	TVAPTECS		45
	AGVETTTPSK		59
	QSNNKYAASSYLSLTPEQWK		43
Ig gamma-3 chain C region, P01860	GPSVFPLAPCSR		44
	TPLGDTTHTCPR		59
	SCDTPPPCPR		52
	VELKTPLGDTTHTCPR		45
	STSGGTAALGCLVK		75
	NQVSLTCLVK		38
Immunoglobulin lambda-like polypeptide 5, B9A064	SYSCQVTHEGSTVEK		60
	VTVLGQPK		38
	SYSCQVTHEGSTVEKTVAPTECS		50
	YAASSYLSLTPEQWK		38
	TVAPTECS		45
	QSNNKYAASSYLSLTPEQWK		43
Ig kappa chain V-III region SIE, P01620	LLIYGASSR		53
	EIVLTQSPGTLSLSPGER		76
Ig kappa chain V-III region B6, P01619	EIVLTQSPGTLSLSPGQR		31
	EIVLTQSPGTLSLSPGER		76

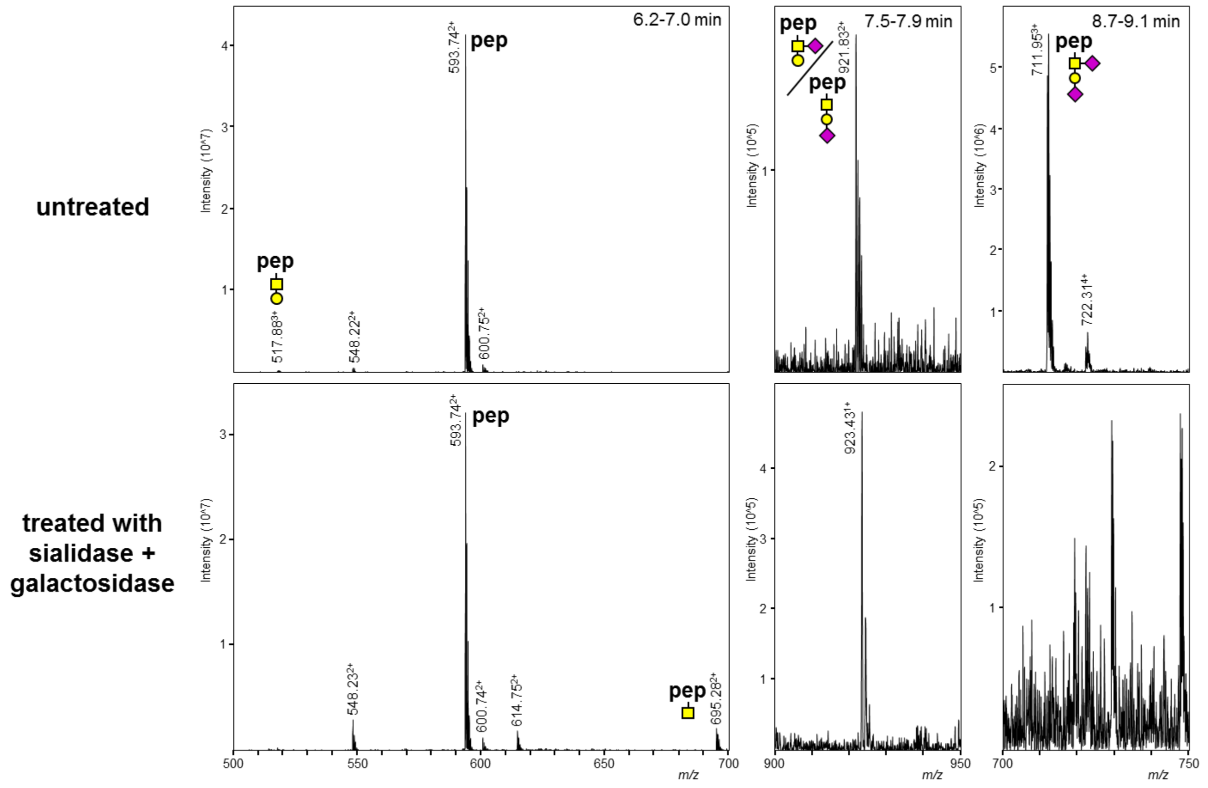

Supplemental Figure S2. NanoLC-ESI-IT-MS spectra showing tryptic O-glycopeptides before and after exoglycosidase digestion. Three mass spectra are shown from time windows during which non-sialylated, monosialylated and disialylated O-glycopeptides eluted. Pep = peptide; yellow square = *N*-acetylgalactosamine, yellow circle = galactose; purple diamond = *N*-acetylneuraminic acid.

Supplemental Table S4. Data generated by nanoLC-ESI-IT-MS(/MS) with CID fragmentation of trypsin-, proteinase K- and chymotrypsin-generated O-glycopeptides identified in various IgG samples. The observed *m/z* of the monoisotopic peak of each (glyco)peptide in MS1 was compared to the theoretical value. If the glycopeptide was fragmented, the observed *m/z* of the monoisotopic peak of the peptide in MS2 is listed; otherwise it is indicated that the glycopeptide was not fragmented (NF) or that the peptide peak was not detected (ND). The glycan structure is denoted using the following symbols: N = *N*-acetylhexosamine; H = hexose; S = *N*-acetylneuraminic acid.

Please see the separate excel file for this table.

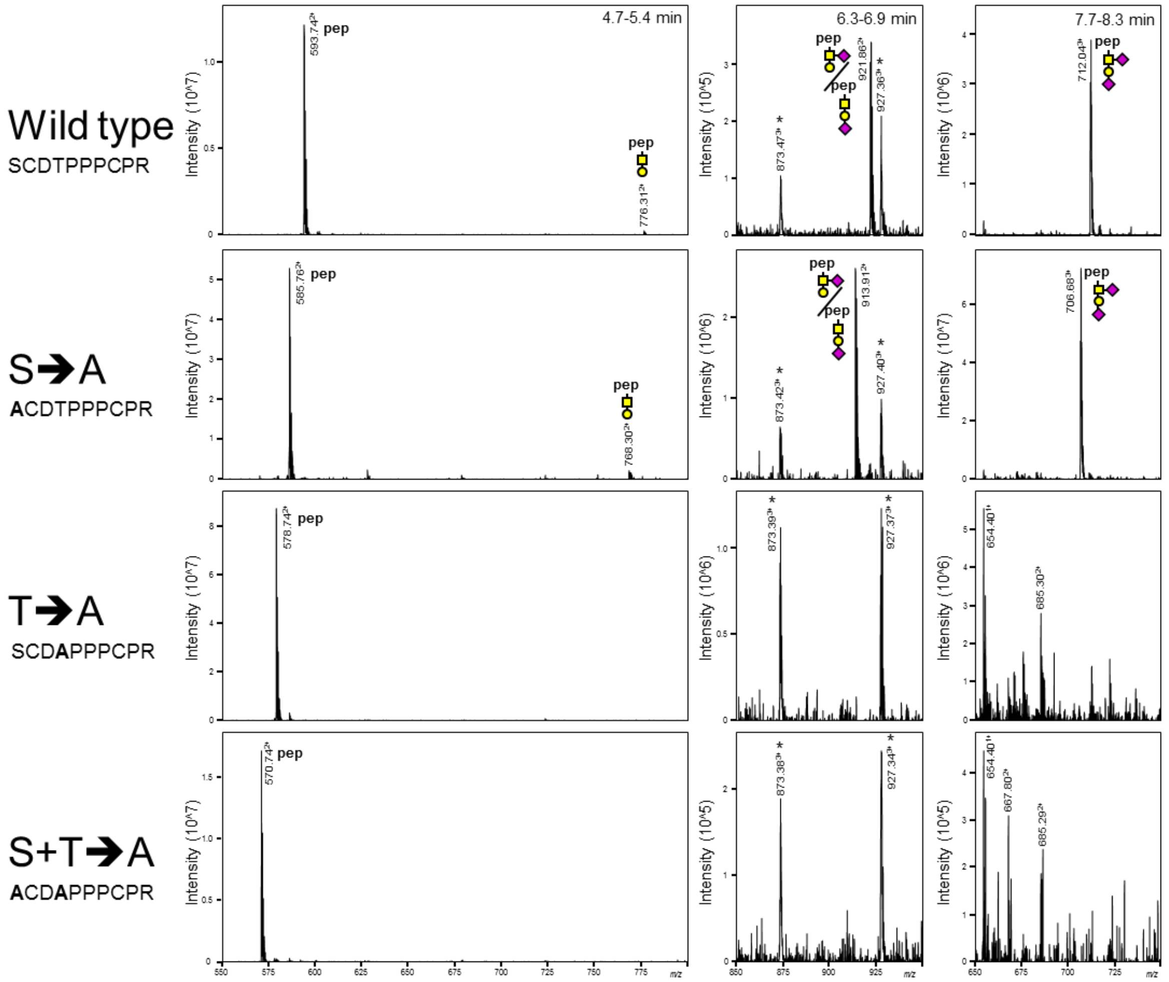

Supplemental Figure S3. NanoLC-ESI-IT-MS spectra showing tryptic O-glycopeptides of wild type IgG3 monoclonal and various hinge mutants. Three mass spectra are shown from time windows during which non-sialylated, monosialylated and disialylated O-glycopeptides eluted. Peaks corresponding to IgG3 N-glycopeptides are marked with an asterisk. Pep = peptide; yellow square = *N*-acetylgalactosamine, yellow circle = galactose; purple diamond = *N*-acetylneuraminic acid.

Supplemental Table S5. A) Relative quantification of IgG3 O-glycosylation based on nanoLC-ESI-IT-MS analysis of tryptic (glyco)peptides from various IgG3 samples (IgG3 purified from six donors (D1-6), IgG3 derived from pooled plasma, and 2 monoclonal IgG3 allotypes). The signal intensities were normalized on the sum of both O-glycopeptides and the corresponding non-glycosylated peptide. The relative abundance and technical variation are based on LC-MS analyses of 4 distinct trypsin digests, each of them measured twice. The values given for glycopeptide NHS are expected to be significantly lower than the actual values, because the triply charged compound overlapped with the acetylated hinge peptide in all samples, leaving only the doubly charged peak for relative quantification. B) Relative quantification of tryptic (glyco)peptides which have been treated with exoglycosidases, trimming all O-glycans down to a single *N*-acetylhexosamine. The relative abundance and standard deviation are based on 2 LC-MS analyses of the same sample. N = *N*-acetylhexosamine; H = hexose; S = *N*-acetylneuraminic acid.

A) Relative quantification of trypsin-generated IgG3 O-glycopeptides

glycan structure	IgG3 sample									
	D1		D2		D3		D4		D5	
	relative abundance	st. dev.	relative abundance	st. dev.	relative abundance	st. dev.	relative abundance	st. dev.	relative abundance	st. dev.
pep	88.9	1.3	87.5	2.2	88.4	2.6	89.0	2.3	88.5	1.7
NH	1.1	0.2	1.2	0.2	1.0	0.2	0.9	0.3	1.0	0.4
NHS	0.3	0.0	0.3	0.1	0.2	0.1	0.3	0.1	0.2	0.0
NHS2	9.8	1.3	11.0	2.0	10.3	2.6	9.8	2.3	10.3	1.8
N2H2S2	0.0	0.0	0.0	0.0	0.0	0.0	0.0	0.0	0.0	0.0
% glyco-sylated	11.1	1.3	12.5	2.2	11.6	2.6	11.0	2.3	11.5	1.7
#NeuAc /glycan	1.8	0.1	1.8	0.0	1.8	0.1	1.8	0.1	1.8	0.1

A) Relative quantification of trypsin-generated IgG3 O-glycopeptides (continued)

glycan structure	IgG3 sample							
	D6		IgG3-pooled plasma		IgG3m(g)		IgG3m(s)	
	relative abundance	st. dev.	relative abundance	st. dev.	relative abundance	st. dev.	relative abundance	st. dev.
pep	88.2	1.6	96.7	0.6	78.1	2.9	81.5	1.8
NH	1.2	0.3	1.2	0.3	0.7	0.2	0.9	0.1
NHS	0.3	0.0	1.3	0.4	0.8	0.1	0.6	0.1
NHS2	10.3	1.5	0.7	0.2	19.4	2.8	16.7	1.7
N2H2S2	0.0	0.0	0.0	0.0	1.0	0.2	0.4	0.1
% glyco-sylated	11.8	1.6	3.3	0.6	21.9	2.9	18.5	1.8
#NeuAc /glycan	1.8	0.0	0.8	0.1	1.9	0.0	1.9	0.0

B) Relative quantification of exoglycosidase-treated trypsin-generated IgG3 O-glycopeptides										
glycan structure	IgG3 sample									
	D1		D2		D3		D4		D5	
	relative abundance	st. dev.	relative abundance	st. dev.	relative abundance	st. dev.	relative abundance	st. dev.	relative abundance	st. dev.
pep	91.2	0.1	89.8	0.7	89.7	1.1	90.4	1.6	91.9	4.0
N	8.8	0.1	10.2	0.7	10.3	1.1	9.6	1.6	8.1	4.0

B) Relative quantification of exoglycosidase-treated trypsin-generated IgG3 O-glycopeptides (continued)								
glycan structure	IgG3 sample							
	D6		IgG3-pooled plasma		IgG3m(g)		IgG3m(s)	
	relative abundance	st. dev.	relative abundance	st. dev.	relative abundance	st. dev.	relative abundance	st. dev.
pep	90.7	1.0	95.0	0.0	85.8	0.1	87.3	0.6
N	9.3	1.0	5.0	0.0	14.2	0.1	12.7	0.6

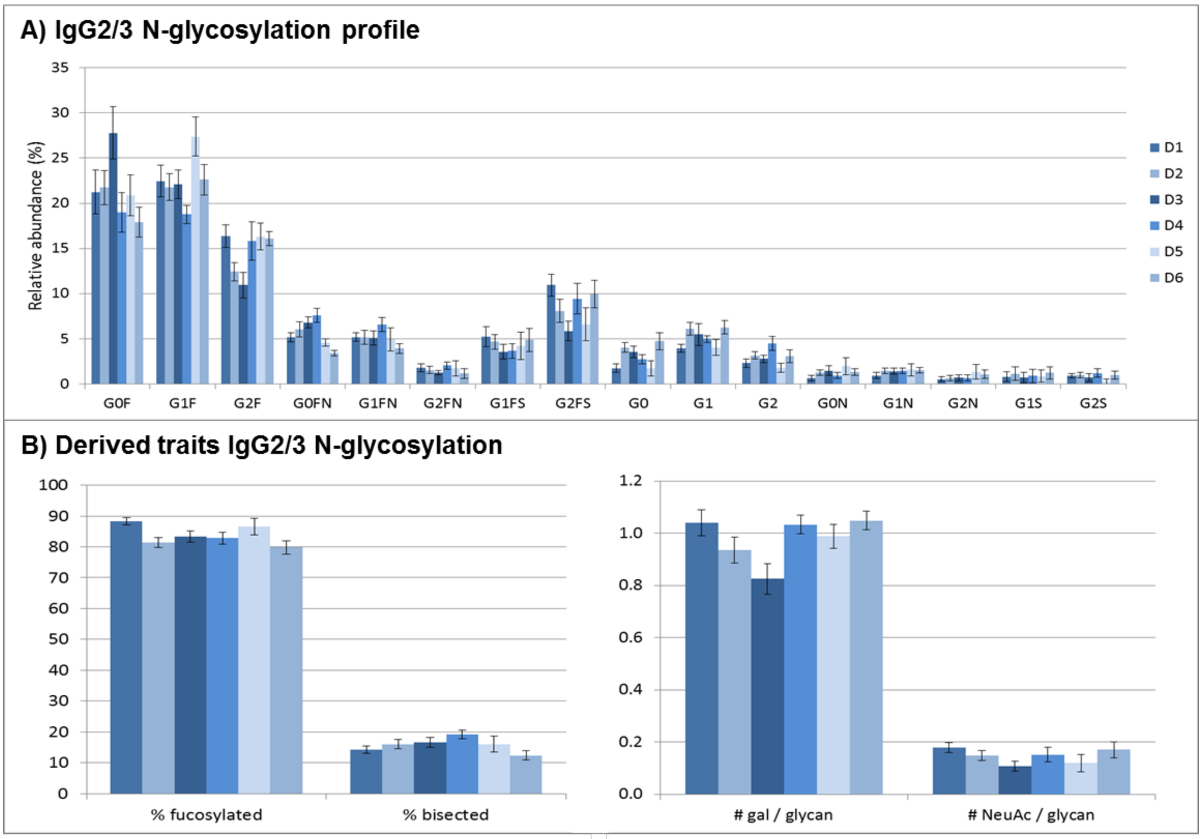

Supplemental Figure S4. A) Fc N-glycosylation profiles of IgG2/3 composed from nanoLC-ESI-IT-MS analysis of tryptic IgG glycopeptides derived from the serum of various donors (D1-6). The sequence of the tryptic peptide covering N297 is the same in IgG3 and IgG2, and thus we cannot distinguish between IgG3 and IgG2 N-glycopeptides. The relative abundance and technical variation are based on LC-MS analyses of 4 distinct trypsin digests, each of them measured twice. Glycopeptides were included if they exhibited a signal-to-noise ratio of over 3 in at least 25% of the samples. The values represented in this figure are listed in Supplemental Table S6. B) Various derived traits were calculated from these profiles: the percentage of fucosylated and bisected glycopeptides and the number of galactoses and *N*-acetylneuraminic acid residues per glycan. G = galactose, F = fucose, N = bisecting *N*-acetylglucosamine, S = *N*-acetylneuraminic acid.

Supplemental Table S6. Fc N-glycosylation profiles of IgG2/3 composed from nanoLC-ESI-IT-MS analysis of tryptic IgG glycopeptides derived from the serum of various donors (D1-6) and pooled plasma. Details related to the analyses used for this data are listed in Supplemental Figure S4. Various derived traits were calculated from these profiles: the percentage of fucosylated and bisected glycans and the number of galactoses and *N*-acetylneuraminic acids per glycan. G = galactose, F = fucose, N = bisecting *N*-acetylglucosamine, S = *N*-acetylneuraminic acid.

glycan structure	IgG3 sample							
	D1		D2		D3		D4	
	relative abundance	st. dev.	relative abundance	st. dev.	relative abundance	st. dev.	relative abundance	st. dev.
G0F	21.2	2.4	21.7	1.9	27.8	2.9	19.0	2.2
G1F	22.5	1.8	21.8	1.5	22.1	1.6	18.8	1.0
G2F	16.4	1.3	12.4	1.0	10.9	1.4	15.8	2.1
G0FN	5.1	0.5	6.0	0.9	6.8	0.6	7.6	0.8
G1FN	5.2	0.5	5.2	0.8	5.1	0.8	6.6	0.8
G2FN	1.8	0.5	1.5	0.4	1.2	0.2	2.0	0.4
G1FS	5.2	1.1	4.7	0.8	3.6	0.8	3.7	0.8
G2FS	10.9	1.2	8.1	1.3	5.9	1.1	9.4	1.7
G0	1.7	0.5	4.0	0.6	3.5	0.6	2.7	0.5
G1	3.9	0.4	6.1	0.7	5.5	1.2	4.9	0.4
G2	2.3	0.5	3.1	0.4	2.8	0.4	4.5	0.8
G0N	0.6	0.3	1.2	0.3	1.5	0.5	0.9	0.3
G1N	0.9	0.4	1.5	0.3	1.4	0.3	1.4	0.3
G2N	0.5	0.3	0.6	0.3	0.7	0.3	0.6	0.3
G1S	0.8	0.5	1.1	0.7	0.7	0.6	0.9	0.7
G2S	0.9	0.2	0.9	0.3	0.7	0.5	1.2	0.5
% fucosylated	88.3	1.2	81.4	1.7	83.3	1.9	82.8	2.0
% bisected	14.2	1.2	16.0	1.5	16.6	1.5	19.2	1.5
# Gal/glycan	1.04	0.05	0.94	0.05	0.83	0.06	1.03	0.04
# NeuAc/glycan	0.18	0.02	0.15	0.02	0.11	0.02	0.15	0.03

glycan structure	IgG3 sample (continued)					
	D5		D6		IgG3-pooled plasma	
	relative abundance	st. dev.	relative abundance	st. dev.	relative abundance	st. dev.
**G0F**	20.9	2.2	17.9	1.7	21.4	1.6
**G1F**	27.4	2.2	22.6	1.7	20.1	1.4
**G2F**	16.3	1.5	16.1	0.8	14.0	1.4
**G0FN**	4.6	0.4	3.4	0.3	6.6	0.7
**G1FN**	4.9	1.3	3.9	0.6	4.9	0.6
**G2FN**	1.7	0.8	1.1	0.5	1.4	0.5
**G1FS**	4.2	1.5	4.9	1.3	4.0	0.8
**G2FS**	6.6	1.8	10.0	1.5	8.8	0.8
**G0**	1.7	0.8	4.7	1.0	4.2	1.0
**G1**	4.0	0.9	6.3	0.8	5.6	0.4
**G2**	1.8	0.5	3.0	0.7	3.4	0.6
**G0N**	2.0	0.9	1.3	0.4	1.4	0.3
**G1N**	1.5	0.7	1.5	0.3	1.4	0.4
**G2N**	1.3	0.8	1.1	0.5	0.8	0.4
**G1S**	0.8	0.7	1.2	0.7	0.8	0.2
**G2S**	0.2	0.3	1.0	0.4	1.1	0.2
**% fucosylated**	86.6	2.7	79.9	2.2	81.3	1.6
**% bisected**	16.0	2.6	12.4	1.6	16.7	1.0
**# Gal/glycan**	0.99	0.05	1.05	0.04	0.96	0.05
**# NeuAc/glycan**	0.12	0.03	0.17	0.03	0.15	0.01

Supplemental Table S7. Relative quantification of O-glycans linked to peptide dimers. Monoclonal IgG3 was digested with trypsin without reduction alkylation, allowing relative quantification of disulfide bridge-linked hinge repeat peptides from LC-MS analysis, listed under 'observed value'. The expected % of peptide dimers with one or two O-glycans was also calculated from the single peptide O-glycan percentages (Suppl. Table S5), assuming randomly distributed occupation of the O-glycosylation sites. The glycan structures are denoted using the following symbols: N = *N*-acetylhexosamine; H = hexose; S = *N*-acetylneuraminic acid.

G3m(g)					
**O-glycan(s)**	**% glycosylation single peptide**	**% glycosylation dimer**			
		**calculation expected value**	**expected value**	**observed value**	**Δ**
**-**	78.13	= 100 * (0.7813 * 0.7813)	61.0	59.3	-1.7
**1(NH)**	0.71	= 100 * (0.0071 * 0.7813 * 2)	1.1	0.4	-0.7
**1(NHS)**	0.81	= 100 * (0.0081 * 0.7813 * 2)	1.3	2.8	+1.6
**1(NHS2)**	19.38	= 100 * (0.1938 * 0.7813 * 2)	30.3	33.9	+3.6
**1(N2H2S2)**	0.98	= 100 * (0.0098 * 0.7813 * 2)	1.5	2.5	+1.0
**2(NHS2)**	-	= 100 * (0.1938 * 0.1938)	3.8	1.0	-2.7
**G3m(s)**					
**O-glycan(s)**	**% glycosylation single peptide**	**% glycosylation dimer**			
		**calculation expected value**	**expected value**	**observed value**	**Δ**
**-**	81.47	= 100 * (0.8147 * 0.8147)	66.4	65.4	-0.9
**1(NH)**	0.90	= 100 * (0.0090 * 0.8147 * 2)	1.5	0.5	-1.0
**1(NHS)**	0.59	= 100 * (0.0059 * 0.8147 * 2)	1.0	2.3	+1.3
**1(NHS2)**	16.67	= 100 * (0.1667 * 0.8147 * 2)	27.2	29.7	+2.6
**1(N2H2S2)**	0.37	= 100 * (0.0037 * 0.8147 * 2)	0.6	1.6	+1.0
**2(NHS2)**	-	= 100 * (0.1667 * 0.1667)	2.8	0.4	-2.3

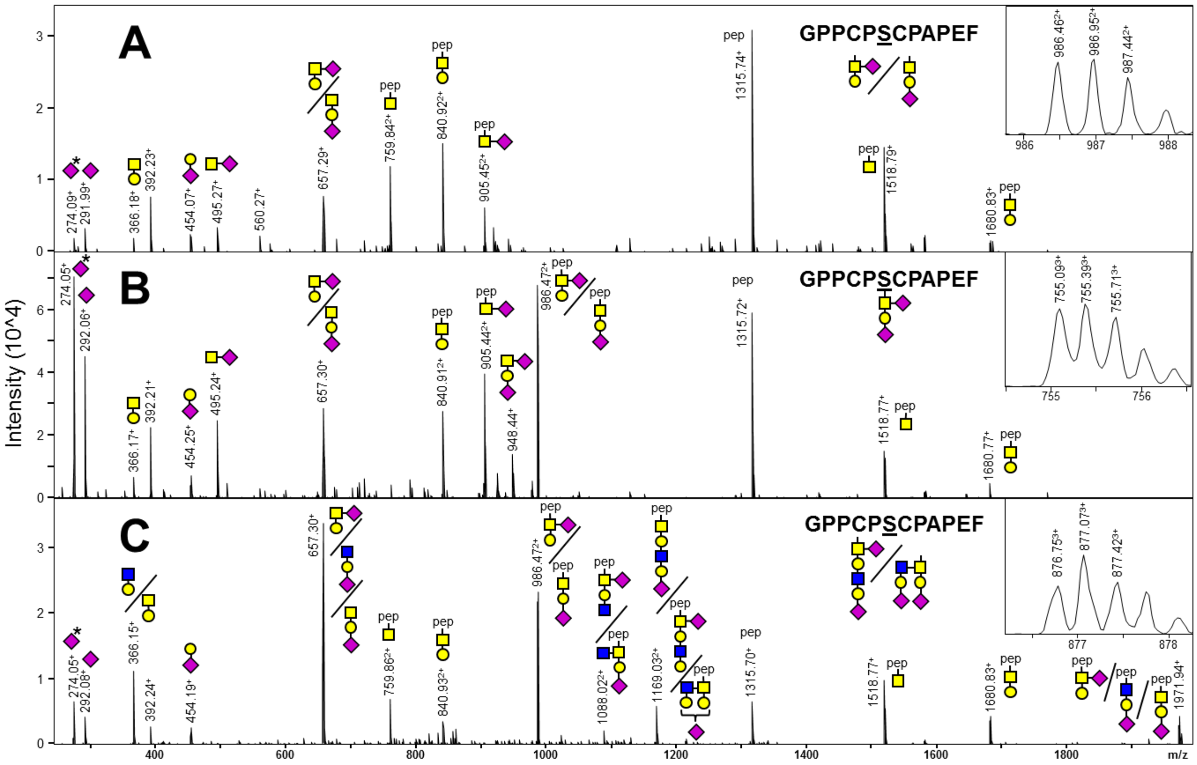

Supplemental Figure S5. NanoLC-ESI-IT-CID spectra showing fragmentation of the proteinase K-generated glycopeptide GPPCPSCPAPEF belonging to an IgG4 Fc construct (IgG4-Fc-V397MA). The following glycan structures were observed: A) a monosialylated core 1 type O-glycan; B) a disialylated core 1 type O-glycan; C) a disialylated O-glycan with an *N*-acetyllactosamine (GlcNAc + Gal). This structure interpretation is partially based on literature, since mass spectrometry cannot distinguish between different types of hexoses and *N*-acetylhexosamines. The MS1 precursor peak of each MS2 spectrum is shown in the right-upper corner. Pep = peptide; yellow circle = galactose; yellow square = *N*-acetylgalactosamine; blue square = *N*-acetylglucosamine; purple diamond = *N*-acetylneuraminic acid.
